# Glycosyl Coumarins as Selective Inhibitors of Tumor-Associated Carbonic Anhydrase IX and XII: Synthesis, Structure–Activity Relationships, and Molecular Modeling

**DOI:** 10.3390/ijms27083659

**Published:** 2026-04-20

**Authors:** Macarena S. Le Pors, Ignacio Aznar, Simone Giovannuzzi, Claudiu T. Supuran, Martin J. Lavecchia, Pedro A. Colinas

**Affiliations:** 1 CEDECOR (UNLP-CICBA), Departamento de Química, Facultad de Ciencias Exactas, Universidad Nacional de La Plata, 47 y 115, La Plata 1900, Argentina; macarenalepors@quimica.unlp.edu.ar (M.S.L.P.); ignacioaznar@quimica.unlp.edu.ar (I.A.); 2 Section of Pharmaceutical Chemistry, NEUROFARBA Department, Università Degli Studi di Firenze, via Ugo Schiff 6, Sesto Fiorentino, 50019 Florence, Italy; simone.giovannuzzi@unifi.it (S.G.); claudiu.supuran@unifi.it (C.T.S.); 3 CEQUINOR (UNLP-CONICET, CCT-La Plata, Associated with CIC), Departamento de Química, Facultad de Ciencias Exactas, Universidad Nacional de La Plata Blvd. 120, La Plata 1465, Argentina

**Keywords:** carbohydrates, anticancer agents, molecular dynamics simulations

## Abstract

Coumarins represent a distinctive class of non-classical carbonic anhydrase inhibitors that interact with the entrance region of the catalytic pocket rather than directly coordinating the catalytic Zn^2+^ ion. In this study, a series of glycosylated coumarins was synthesized through a copper-catalyzed multicomponent reaction involving propargyl glycosides, salicylaldehyde, and tosyl azide, providing efficient access to iminocoumarin-based glycosides derived from natural carbohydrates. The inhibitory activity of the synthesized compounds was evaluated against human carbonic anhydrase isoforms I, II, IX, and XII using a stopped-flow CO_2_ hydrase assay. The compounds showed negligible inhibition of the cytosolic isoforms *h*CA I and *h*CA II, while displaying moderate activity toward the tumor-associated isoforms *h*CA IX and *h*CA XII, with Ki values ranging from 12.9 to 41.8 μM. Among the series, 6-*O*-(2*H*-chromene-2-one-3-yl-methyl)-D-galactopyranose (**10a**) emerged as the most potent inhibitor of *h*CA IX and XII. Structure–activity relationship analysis indicated that deprotected glycosyl derivatives exhibit improved inhibitory activity compared to protected analogues. To rationalize these observations, molecular docking followed by molecular dynamics simulations and MM-GBSA binding free energy calculations were performed for both anomeric forms of compound **10a**. The computational results revealed a clear preference for the β-anomer, particularly in *h*CA IX and XII, where favorable interactions with catalytic threonine residues and isoform-specific aromatic residues stabilize the ligand within the active-site entrance. These findings provide a molecular explanation for the experimentally observed selectivity and highlight glycosyl coumarins as potential starting points for further optimization toward selective inhibitors of tumor-associated carbonic anhydrases.

## 1. Introduction

Coumarins (2*H*-1-benzopyran-2-ones) constitute a structurally diverse family of benzopyrone derivatives widely distributed in plants, fungi, and microorganisms. The coumarin scaffold is considered a privileged structure in medicinal chemistry due to its synthetic accessibility, structural tunability, and broad spectrum of biological activities [1]. Structural diversification through substitution or ring fusion (e.g., furanocoumarins, pyranocoumarins, and glycosylated derivatives) enables modulation of physicochemical properties and target selectivity.

Natural and synthetic coumarins display a wide array of pharmacological activities, including anticoagulant, antimicrobial, antiviral, antioxidant, anti-inflammatory and anticancer effects [1,2]. Among their validated molecular targets are the human carbonic anhydrases (*h*CAs, EC 4.2.1.1), zinc metalloenzymes that catalyze the reversible hydration of carbon dioxide to bicarbonate and a proton. While cytosolic isoforms such as *h*CA I and *h*CA II are ubiquitously expressed and essential for physiological acid–base balance, the transmembrane isoform *h*CA IX is strongly upregulated in hypoxic tumors and plays a key role in extracellular acidification, tumor progression, invasion and metastasis [3]. Consequently, selective inhibition of *h*CA IX has emerged as a promising strategy for the development of anticancer agents [4].

Coumarins represent a mechanistically distinct class of carbonic anhydrase inhibitors. Unlike classical sulfonamides, which coordinate directly to the catalytic Zn(II) ion, coumarins act through a non-classical, enzyme-activated mechanism. Mechanistic studies demonstrated that coumarins behave as prodrug or “suicide” inhibitors: the esterase activity of the enzyme hydrolyzes the lactone ring to generate 2-hydroxycinnamic acid derivatives, which subsequently bind at the entrance of the active-site cavity rather than interacting directly with the zinc center [5,6]. This mechanism explains the characteristic time-dependent inhibition observed in kinetic assays and highlights the distinct binding mode of coumarin-based inhibitors compared with classical carbonic anhydrase inhibitors.

Structural and kinetic investigations have provided insight into the inhibition of *h*CA II and tumor-associated *h*CA IX by coumarins. X-ray crystallography and molecular modeling studies revealed that hydrolyzed coumarin derivatives occupy the rim of the active-site cavity, interacting with residues located at the entrance of the catalytic pocket [5,7]. Importantly, this region exhibits significant sequence variability among CA isoforms. As a result, coumarin derivatives can achieve isoform selectivity by exploiting structural differences in the active-site entrance rather than the highly conserved zinc-binding region. Indeed, many coumarin derivatives show weak inhibition of the ubiquitous cytosolic isoform *h*CA II while displaying significantly stronger inhibition of the tumor-associated isoform *h*CA IX [6,8,9,10,11].

An important subclass of coumarin-based carbonic anhydrase inhibitors comprises glycosyl (carbohydrate-decorated) coumarins. The incorporation of carbohydrate moieties can improve aqueous solubility, modulate steric interactions at the active-site entrance, and enhance isoform selectivity. Touisni et al. reported a series of glycosyl coumarins that selectively inhibit *h*CA IX and XII over *h*CA I and II and significantly attenuate the growth of primary breast tumor xenografts, providing in vivo evidence that selective inhibition of tumor-associated carbonic anhydrases by coumarin derivatives can translate into antitumor activity [12]. Subsequent studies have explored additional coumarin–carbohydrate conjugates prepared using modular synthetic approaches, confirming that the incorporation of sugar units can contribute to improved selectivity profiles toward tumor-associated CA isoforms [13].

From a synthetic perspective, glycosyl coumarins can be prepared using several complementary methodologies. Classical *O*-glycosylation of hydroxycoumarins, particularly 7-hydroxycoumarins, with protected glycosyl donors such as glycosyl halides, thioglycosides, or trichloroacetimidates under Lewis acid activation represents one of the most widely employed strategies [14]. These approaches allow stereochemical control of the glycosidic bond through protecting-group design and neighboring-group participation. In addition to direct glycosylation methods, Cu(I)-catalyzed azide–alkyne cycloaddition (CuAAC) has emerged as a powerful modular strategy for the preparation of triazole-linked carbohydrate–coumarin conjugates, enabling independent diversification of both the coumarin core and the carbohydrate fragment [13,15]. Alternative synthetic routes have also been explored for the preparation of glycosyl coumarin derivatives. For example, *C*-glycosyl coumarins, in which the sugar is attached through a carbon–carbon bond rather than an *O*-glycosidic linkage, can be synthesized through Friedel–Crafts-type glycosylations, transition-metal-catalyzed cross-coupling reactions, or domino condensation strategies [16].

As previously noted, the chemical space and inhibitory profiles of glycosylated coumarins against tumor-associated carbonic anhydrase isoforms remain largely underexplored. Herein, we describe the synthesis of a small library of glycosides bearing the coumarin moiety via a copper-catalyzed multicomponent reaction employing propargyl glycosides derived from natural carbohydrates. The synthesized compounds were evaluated as inhibitors of human carbonic anhydrase isoforms I, II, IX, and XII, and the resulting structure–activity relationships were analyzed. Furthermore, molecular docking, molecular dynamics simulations, and MM-GBSA binding free energy calculations were performed to rationalize the experimentally observed inhibition profiles and to gain insight into the molecular determinants governing isoform selectivity.

## 2. Results and Discussion

**Scheme 1:**
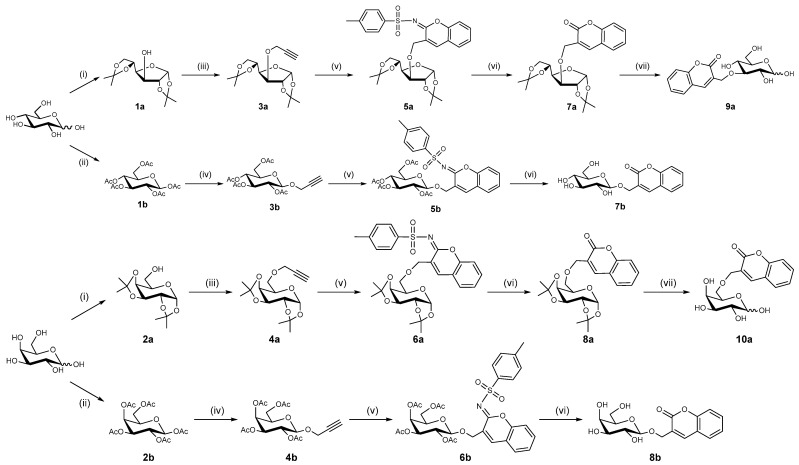
Reagents and conditions: (i) I_2_, acetone, rt, 48 h (78–80%); (ii) NaAcO, Ac_2_O, reflux, 2.5 h (45–50%); (iii) propargyl bromide, *n*-Bu_4_NHSO_4_, NaOH 50%, CH_2_Cl_2_, rt, 5 h (60–65%); (iv) propargyl alcohol, BF_3_·Et_2_O, K_2_CO_3_, CH_2_Cl_2_, N_2_, rt, 24 h (64–76%); (v) salicylaldehyde, TsN_3_, CuI, NEt_3_, THF, N_2_, rt, 13 h (44–97%); (vi) NaMeO, MeOH, H_2_O, rt, 12 h (25–98%); (vii) TFA, H_2_O, rt, 24 h (69–78%).

Our synthetic strategy for glycosyl coumarins relies on a well-established copper-catalyzed multicomponent reaction involving propargyl glycosides, [17] which were prepared in two steps from natural carbohydrates (Scheme 1). First, the sugars were protected with isopropylidene or acetate groups. The protected carbohydrates were then reacted with propargyl bromide under phase-transfer catalysis conditions, affording glycosides **3a–b** and **4a–b** in very good yields. These propargyl glycosides were subsequently engaged in the multicomponent reaction by combining **3a–b**, **4a–b**, salicylaldehyde, and tosyl azide in THF with triethylamine as base and CuI as catalyst. This one-pot transformation facilitates the formation of the iminocoumarin core while simultaneously installing the glycosyl moiety via the generated triazolyl copper intermediate [18]. This cascade process provides an efficient route to access structurally diverse glycosides, while circumventing the need for the isolation of reactive azide-containing intermediates. Treatment of iminocoumarins **5a** and **6a** with NaOMe in MeOH gave protected glycosides **7a** and **8a**. Subsequent treatment with aqueous TFA afforded fully deprotected glycosides **9a** and **10a**. In contrast, iminocoumarins **5b** and **6b** under basic conditions yielded de-*O*-acetylated glycosyl coumarins **7b** and **8b**. This synthetic approach afforded a small library of both protected and deprotected glycosides bearing a coumarin moiety at various positions on the carbohydrate scaffold.

The inhibitory activity of the synthesized glycosyl coumarins (**7a**–**10a**) was evaluated against the human carbonic anhydrase isoforms *h*CA I, *h*CA II, *h*CA IX, and *h*CA XII using a stopped-flow CO_2_ hydrase assay (Table 1).

A clear isoform selectivity profile emerged from the inhibition data. All compounds showed negligible inhibitory activity toward the cytosolic isoforms *h*CA I and *h*CA II, with Ki values higher than 100 μM. This behavior is consistent with the well-known inhibition mechanism of coumarins, which act as prodrug inhibitors that are hydrolyzed by the esterase activity of carbonic anhydrases and subsequently bind at the entrance of the catalytic cavity rather than coordinating the Zn^2+^ ion directly. Because the entrance region of the catalytic pocket exhibits significant variability among CA isoforms, coumarin derivatives frequently display selectivity toward tumor-associated isoforms such as *h*CA IX and XII.

In contrast to the lack of inhibition observed for *h*CA I and II, all compounds displayed moderate inhibitory activity against the tumor-associated isoforms *h*CA IX and *h*CA XII. The Ki values for *h*CA IX ranged from 12.9 to 41.8 μM, while those for *h*CA XII ranged from 19.6 to 35.6 μM. Among the series, compound **10a** emerged as the most potent inhibitor of *h*CA IX, exhibiting a Ki value of 12.9 μM. Compound **9a** also showed relatively improved activity (Ki = 22.3 μM), suggesting that the presence of fully deprotected glycosyl moieties may enhance interactions with the enzyme active site.

A comparison between protected and deprotected derivatives within this limited series reveals an interesting trend. Compounds **7a** and **8a**, which still bear protecting groups on the carbohydrate moiety, generally display weaker inhibition of *h*CA IX than the corresponding deprotected analogues **9a** and **10a**. This observation suggests that both the degree of carbohydrate deprotection and the overall hydrophilicity of the molecule contribute to improved binding interactions at the catalytic pocket.

The influence of the carbohydrate substitution pattern is also reflected in the inhibition data. While compounds **7b–8b**, incorporating coumarin at the anomeric center, show relatively similar inhibitory profiles toward *h*CA IX (Ki values between ~38 and 42 μM), structural modification leading to compounds **9a** and **10a** significantly improves potency, particularly for *h*CA IX.

Regarding inhibition of *h*CA XII, the compounds display slightly more uniform activity across the series, with Ki values ranging from 19.6 to 35.6 μM. Compound **10a** again shows the best inhibitory potency (Ki = 19.6 μM), indicating that the structural features beneficial for *h*CA IX inhibition may also contribute to inhibition of *h*CA XII.

Overall, the SAR analysis indicates that:

1. Glycosyl coumarins exhibit strong selectivity toward tumor-associated carbonic anhydrase isoforms over cytosolic isoforms.
2. Deprotected carbohydrate moieties improve inhibitory potency, likely due to enhanced hydrogen-bonding interactions and increased hydrophilicity.
3. Subtle variations in carbohydrate substitution and protection patterns significantly influence the inhibition of *h*CA IX and XII.

Although the potency of the synthesized compounds is significantly lower than that of the reference inhibitor acetazolamide, which shows nanomolar inhibition, the observed isoform selectivity is notable and supports the potential of glycosyl coumarins as scaffolds for further development of selective carbonic anhydrase inhibitors targeting tumor-associated isoforms.

To further rationalize the experimentally observed SAR trends, molecular docking followed by molecular dynamics (MD) simulations and MM-GBSA binding free energy calculations were performed for the most active compound, **10a**, considering both its α and β anomeric forms. As coumarins inhibit carbonic anhydrase via an enzyme-catalyzed hydrolysis process that transforms the parent lactone into a 2-hydroxycinnamic acid derivative, molecular docking studies were performed using the hydrolyzed form of both anomers of glycoside **10a** [6].

**Figure 1:**
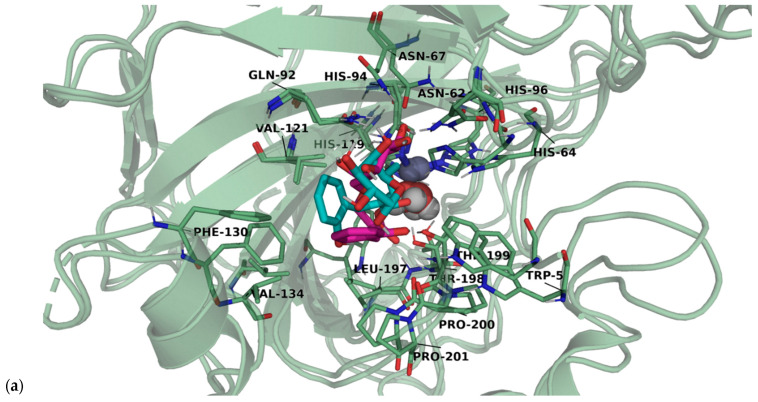

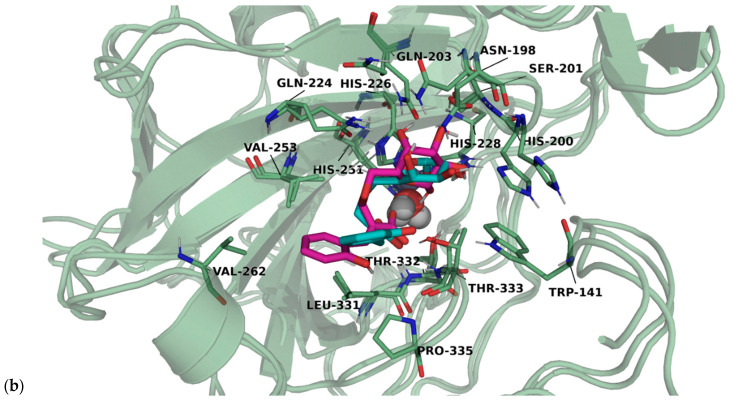
Representative binding modes of the **10a** anomers within the (**a**) *h*CA II and (**b**) *h*CA IX active sites. Structural superposition of the last frame extracted from the MD trajectories. The α-**10a** is shown in cyan, while the β-**10a** is represented in pink. The protein backbone and key catalytic residues are shown as ribbons/sticks, and the catalytic Zinc ion is depicted as a gray sphere. Figure generated using PyMOL 3.1.0. *h*CA I and *h*CA XII complexes are shown in Figure S5.

The top-ranked docking poses for each *h*CA–ligand complex were selected as the starting configurations for the subsequent MD simulations. The structural stability and equilibration of the simulated *h*CA–ligand complexes were monitored by calculating the Root Mean Square Deviation (RMSD) for both the protein backbone and the ligand relative to the initial equilibrated structures. The RMSD profiles (Figures S1–S4) demonstrate that all systems, except *h*CA I/α-**10a**, rapidly reached structural convergence, maintaining stable plateaus throughout the production trajectories. This stability ensures that the sampled conformational ensembles are reliable for the subsequent MM-GBSA energy decomposition and protein–ligand interaction fingerprint analyses. The complexes between anomers of **10a** and *h*CAs are shown in Figure 1. In the case of the *h*CA I complex with the α-**10a**, a notable conformational reorganization was observed during the equilibration phase. As illustrated in Figure S7, the ligand underwent a significant rotation relative to the initial docking pose before reaching a stable plateau in its final equilibrated state (cyan).

The MM-GBSA binding free energy calculations (Table 2) reveals a clear preference for the β-anomer across most isoforms, with the most pronounced effects observed for the *h*CA IX and XII. Specifically, the *h*CA XII/β-**10a** complex exhibited the highest affinity.

To rationalize the differences in binding affinity, we combined per-residue MM-GBSA decomposition with dynamic interaction fingerprints mapped onto the sequence alignment. This approach allowed us to identify isoform-specific hotspots associated with the observed stereoselectivity.

**Figure 2:**
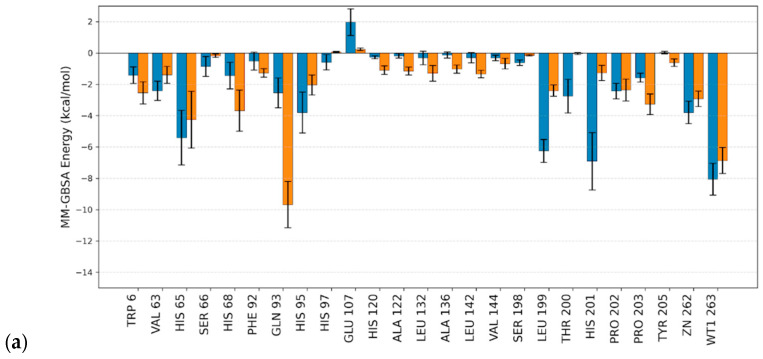

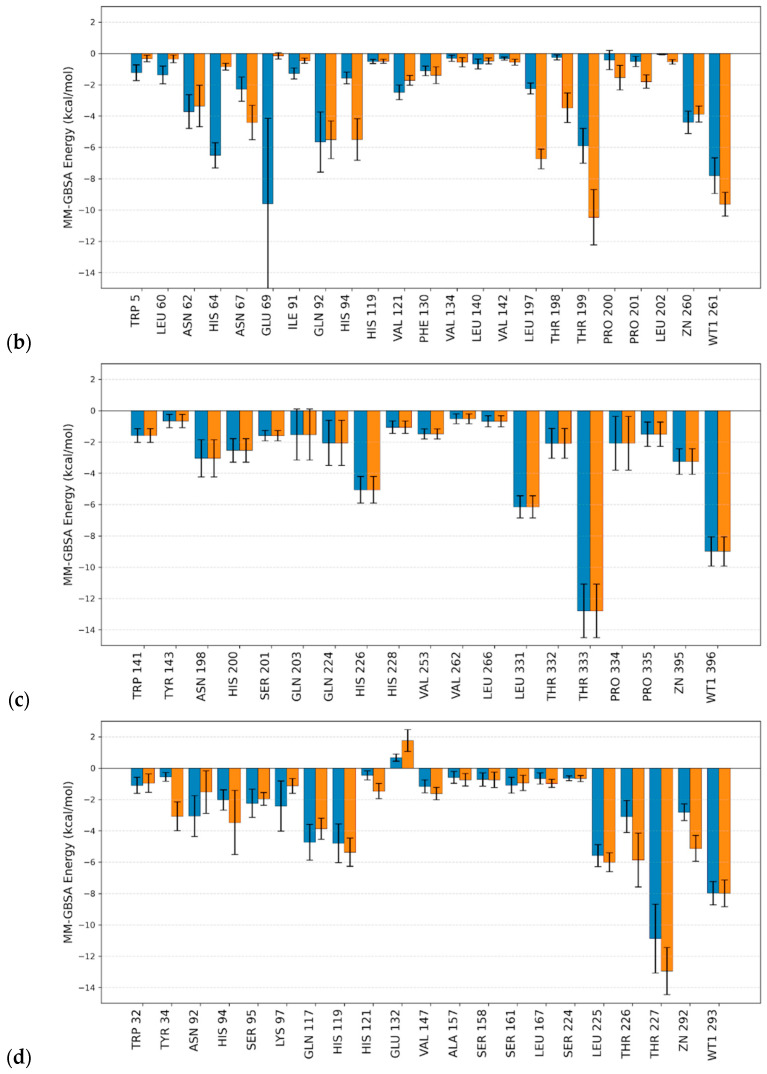
Per-residue MM-GBSA binding energy decomposition. Comparison between the α-anomer (blue) and β-anomer (orange) interaction with (**a**) *h*CA I, (**b**) *h*CA II, (**c**) *h*CA IX, and (**d**) *h*CA XII. Only residues with an absolute interaction energy contribution higher than 0.5 kcal mol^−1^ for at least one of the anomers are displayed.

Per-residue energy decomposition analysis, see Figure 2, reveals that the catalytic threonine serves as a critical stabilization hotspot within the active site across most isoforms. In the membrane-associated *h*CA IX and *h*CA XII, the homologous residues THR333 and THR227, respectively, show stabilizing contributions, reaching approximately −12.5 kcal mol^−1^. For *h*CA II, the corresponding THR199 maintains a strong but slightly reduced interaction (approximately −10 kcal mol^−1^ and –6 kcal mol^−1^ for the β-anomer and α-anomer, respectively). The structural divergence observed in *h*CA I provides a significant contrast; at this homologous position, the threonine is substituted by HIS201, which exhibits markedly lower interaction energy for the β-anomer. These results indicate that the hydroxyl group of the conserved threonine likely contributes to ligand anchoring through favorable polar interactions. The substitution of this polar anchor for a histidine in *h*CA I likely explains the diminished binding efficiency of the β-anomer in this specific isoform.

The Multiple Sequence Alignment (MSA) identifies a conserved W−X−Y motif (Figure S6), where TYR34 in *h*CA XII aligns with TYR143 in *h*CA IX, TYR7 in *h*CA II, and TYR8 in *h*CA I. This residue exceeds the interaction threshold (>0.5 kcal mol^−1^) only in the membrane-associated isoforms. In *h*CA XII, dynamic fingerprints show that only the β-anomer establishes high-occupancy contacts with TYR34 (95.7% van der Waals interactions (VdW), 84.1% Hydrogen Bond Acceptor (HBA)), Table S1. Similarly, in *h*CA IX, TYR143 contributes more to the stabilization of the β-form compared to the α-form. This Tyr does not meet the interaction threshold in the cytosolic *h*CA I and II isoforms. These observations are consistent with a pocket geometry that favors the β-anomer orientation in *h*CA IX and XII.

The binding pose of the β-anomer within the *h*CA I active site is primarily driven by a high-occupancy interaction with GLN93. This residue establishes a robust hydrogen-bonding and van der Waals network (100% occupancy), effectively anchoring the glycosyl moiety and shifting the ligand toward this hydrophilic sub-pocket. A structurally equivalent interaction is observed in *h*CA II with GLN92, although it contributes to the stabilization of both anomers with varying intensities. In contrast, while homologous contacts are maintained with GLN224 in *h*CA IX and GLN117 in *h*CA XII, their energetic contributions are of a lower magnitude compared to the cytosolic isoforms. Notably, these interactions in the membrane-associated *h*CAs are insufficient to induce the significant structural displacement of the glycosyl moiety.

Furthermore, *h*CA II exhibits an important interaction hotspot with GLU69, which specifically stabilizes the α-anomer. This interaction is characterized by significant energetic fluctuation, as evidenced by the large standard deviation in the MM-GBSA profile. Sequence alignment confirms that this glutamic acid residue is an isoform-specific feature of *h*CA II.

Notably, the MM-GBSA per-residue energy decomposition for *h*CA I reveals a repulsive energetic contribution of approximately +2 kcal mol^−1^ associated with residue GLU107, specifically impacting the α-**10a**. A similar behavior is observed in *h*CA XII for the homologous residue GLU132, which likewise imposes an electrostatic penalty that destabilizes both anomers.

Furthermore, the interaction with the catalytic water molecule, identified as WT1, was found to be a critical and invariant stabilizer across all studied complexes. The energetic contribution of WT1 remains remarkably consistent, providing a stabilization of approximately −8 kcal mol^−1^ regardless of the ligand’s anomer configuration or the specific *h*CA isoform. 

In addition, the computational results indicate that the glycosyl moiety plays a critical role in directing ligand orientation within the active-site entrance. The presence of free hydroxyl groups in the deprotected derivatives facilitates the formation of hydrogen-bonding networks with residues located in the hydrophilic region of the pocket as well as with the catalytic water molecule (WT1), which contributes consistently to the stabilization of the ligand across all complexes. Taken together, these findings provide a molecular-level explanation for the SAR trends observed experimentally, particularly the improved activity of compound **10a** and the enhanced potency of deprotected glycosyl derivatives toward *h*CA IX and *h*CA XII.

It is important to note that the present study is based on a relatively small compound library and a limited panel of carbonic anhydrase isoforms. Therefore, the conclusions regarding structure–activity relationships should be interpreted within this context. In addition, cytotoxicity and toxicity evaluations were beyond the scope of this work and will be addressed in future studies.

## 3. Material and Methods

### 3.1. General Procedure for Carbohydrate Acetylation

In a double neck round bottom flask, sodium acetate (1.1 mmol) was dissolved in acetic anhydride (13.34 mmol). Solution was heated until reflux, and then the appropriate carbohydrate (1 mmol) was slowly added. The reaction mixture was stirred in reflux for 30 min and then was left to cool down to room temperature, after which it was poured into a round bottom flask filled with crushed ice. The suspension was stirred for 2 h in an ice bath. Obtained precipitate was filtered and rinsed with ice cold water. The crude white solid was dried in vacuo and then crystallized from ethanol [19].

1,2,3,4,6-Penta-*O*-acetyl-*β*-D-glucopyranose (**1b**). Yield: 50%. White crystals. Mp: 131–132 °C.

1,2,3,4,6-Penta-*O*-acetyl-*β*-D-galactopyranose (**2b**). Yield: 45%. White crystals. Mp: 128–128.5 °C.

### 3.2. General Procedure for the Synthesis of O-Propargyl Glycosides

The corresponding pentaacetylated carbohydrate (2 mmol) was dissolved in 10 mL of anhydrous dichloromethane. With the solution under N_2_ atmosphere and in an ice-cold bath, propargyl alcohol (3 mmol) was added, followed by the addition of boron trifluoride etherate (3.6 mmol) drop by drop. The mixture was stirred for 24 h at room temperature. After said time, anhydrous potassium carbonate (400 mg) was added and the mixture stirred for 30 min. The precipitate was filtered off, and remaining solution was washed with water 3 × 3 mL and dried with sodium sulfate. Solution was concentrated under reduced pressure to obtain crude product [19]. Glucose derivative was crystallized from ethanol, obtaining white crystals (mp: 112–114 °C), and galactose derivative was purified with column chromatography on silica gel, *n*-hexane:ethyl acetate (1:1) as the mobile phase, obtaining a yellowish oil.

2,3,4,6-Tetra-*O*-acetyl-1-*O*-propargyl-*β*-D-glucopyranoside (**3b**) was crystallized from ethanol. Overall yield: 64%. White crystals. Mp: 112–114 °C.

2,3,4,6-Tetra-*O*-acetyl-1-*O*-propargyl-*β*-D-galactopyranoside (**4b**) was purified with column chromatography on silica gel using *n*-hexane:ethyl acetate (1:1) as the mobile phase. Overall yield: 76%. Yellowish oil.

### 3.3. General Procedure for the Synthesis of Di-O-Isopropylidene-Protected Glycosides

Iodide (2 mmol) was dissolved in 90 mL of acetone and then the corresponding carbohydrate (10 mmol) was added. The reaction mixture was stirred for 48 h at room temperature. Remaining iodide was removed by reaction with a 10% sodium thiosulfite solution (Na_2_S_2_O_2_); the solution was added until the reddish color of the reaction mixture was gone and the white precipitate was filtered off. Reaction mixture was concentrated under reduced pressure, the crude product was extracted with dichloromethane 4 × 10 mL, and the organic phases were washed with water 2 × 10 mL, 10 mL of 10% sodium chloride solution, and 10 mL of water. Remaining organic phases were dried with sodium sulfate and concentrated. Crude product was purified with column chromatography on silica gel and *n*-hexane:ethyl acetate (1:1) as the mobile phase [20].

1,2:5,6-Di-*O*-isopropylidene-*α*-D-glucofuranose (**1a**). Yield: 78%. White solid. Mp: 109–110 °C.

1,2:3,4-Di-*O*-isopropylidene-*α*-D-galactopyranose (**2a**). Yield: 79.7%. Yellowish oil.

### 3.4. General Procedure for the Synthesis of Propargyl Diisopropylidene Glucofuranose and Galactopyranose

To a solution of the corresponding di-*O*-isopropylidene glycoside (4 mmol) in 8.4 mL of dichloromethane, propargyl bromide (6 mmol) and tetrabutylammonium hydrogen sulfate (0.4 mmol) were added. With the mixture inside an ice-cold bath, 8.4 mL of 50% sodium hydroxide solution was slowly added. After 5 h of stirring at room temperature, extractions were made with dichloromethane 4 × 5 mL, and organic phases were washed with water 3 × 5 mL, 5 mL of 10% sodium chloride solution, and 5 mL of water. Combined organic phases were dried with sodium sulfate and concentrated under reduced pressure to obtain crude products. Purifications were conducted with column chromatography on silica gel, with *n*-hexane:ethyl acetate (9:1) as eluent [21]. Glucose derivative was obtained as an orange oil, and galactose derivative was obtained as a white solid (mp: 56–58 °C).

1,2:5,6-Di-*O*-isopropylidene-1-*O*-propargyl-*α*-D-glucofuranose (**3a**). Yield: 60%. Orange oil.

1,2:3,4-Di-*O*-isopropylidene-1-*O*-propargyl-*α*-D-galactopyranose (**4a**). Yield: 64.9%. White solid. Mp: 56–58 °C.

### 3.5. General Procedure for the Synthesis of Tosyl Azide

To a solution of tosyl chloride (5 mmol) in 9.1 mL of acetone in an ice-cold bath, a solution of sodium azide (7.5 mmol) in 2.9 mL of water was added drop by drop. The reaction mixture was stirred for 16 h at room temperature then concentrated under reduced pressure to remove acetone. Extractions were made with dichloromethane 2 × 2 mL, and organic phases were washed with water 2 × 2 mL, 5% sodium carbonate solution 2 × 2 mL, and water 2 × 2 mL. Combined organic phases were dried over magnesium sulfate and concentrated to afford a colorless oil, which solidifies under storage around 4 °C, obtaining colorless crystals. Solid was dried and utilized without further purification [22].

### 3.6. General Procedure for the Synthesis of Glycosyl Iminocoumarin

Corresponding propargyl glycoside (0.5 mmol), salicylaldehyde (0.55 mmol), tosyl azide (0.6 mmol), and cuprous iodide (0.05 mmol) were dissolved in 2.5 mL of tetrahydrofuran, and the mixture was stirred under N_2_ atmosphere. After 1 h, triethylamine (1 mmol) was added to the mixture, and it was left to stir for 12 h at room temperature. The mixture is concentrated under reduced pressure, and crude product is dissolved in dichloromethane. Solution is washed twice with a 5% ammonium chloride solution, saturated sodium chloride solution, and water. Organic phase is dried over sodium sulfate and concentrated to afford crude product. Purifications were conducted with column chromatography on silica gel, with *n*-hexane:ethyl acetate (7:3) as eluent [16].

1-*O*-[2-(4-Methylphenylsulfonylimino)-2*H*-chromene-3-yl-methyl]-2,3,4,6-tetra-*O*-acetyl-*β*-D-glucopyranoside (**5b**). Yield: 44%. Sticky white solid.

1-*O*-[2-(4-Methylphenylsulfonylimino)-2*H*-chromene-3-yl-methyl]-2,3,4,6-tetra-*O*-acetyl-*β*-D-galactopyranoside (**6b**). Yield: 70%. Sticky white solid.

3-*O*-[2-(4-Methylphenylsulfonylimino)-2*H*-chromene-3-yl-methyl]-1,2:5,6-di-*O*-isopropylidene-*α*-D-glucofuranose (**5a**). Yield: 45.2%. Sticky white solid.

6-*O*-[2-(4-Methylphenylsulfonylimino)-2*H*-chromene-3-yl-methyl]-1,2:3,4-di-*O*-isopropylidene-*α*-D-galactopyranose (**6a**). Yield: 96.7%. Sticky yellowish solid.

### 3.7. General Procedure for Sulfonylimino Hydrolysis and Deacetylation

To a solution of the corresponding glycosyl iminocoumarin (1 mmol) in 10 mL of methanol, a saturated solution of sodium methoxide is added slowly, until mixture’s pH is between 10 and 12. Water (10 mmol) is added, and reaction mixture is stirred for 12 h at room temperature. After reaction completion, pH is adjusted to neutral by the slow addition of Dowex-50-Hydrogen resin. Resin is filtered off, and the mixture is concentrated under reduced pressure. Crude products were purified with column chromatography on silica gel; di-*O*-isopropylidene glycosyl coumarins were purified with *n*-hexane:ethyl acetate (6:4) as eluent, while deacetylated coumarins were purified with dichloromethane:methanol (9:1) as eluent [16].

1-*O*-[2*H*-chromene-2-one-3-yl-methyl]-b-D-glucopyranoside (**7b**). Yield: 25%. Orange sticky solid.

^1^H-NMR (500 MHz, DMSO-d_6_) δ: 8.18 (d, 1H, *J*_4,5_ = 2 Hz, H-4), 7.69 (d, 1H, *J*_5,6_ = 7.6 Hz, H-5), 7.64 − 7.57 (m, 1H, H-7), 7.44 − 7.42 (m, 1H, H-8), 7.38 (td, 1H, *J* = 7.5, 1.1 Hz, H-6), 5.24 (d, 1H, *J* = 4.8 Hz, OH), 5.01 (d, 1H, *J* = 4.8 Hz, OH), 4.97 − 4.87 (m, 1H, OH), 4.69 (dd, 1H, *J* = 15.4 Hz, CH_2_), 4.56 (t, 1H, *J* = 5.9 Hz, OH), 4.50 (dd, 1H, *J* = 15.4 Hz, CH_2_), 4.33 (d, 1H, *J*_1′,2′_ = 7.7 Hz, H-1′), 3.70 (ddd, 1H, *J*_6′a,6′b_ = 11.8 Hz, *J*_6′a,5′_ = 5.9 Hz, H-6′a), 3.46 (dt, 1H, *J*_6′b,6′a_ = 11.6 Hz, *J*_6′b,5′_ = 5.8 Hz, H-6′b), 3.22 − 3.07 (m, 4H, H-2′, H-3′, H-4′, H-5′).

^13^C-NMR (126 MHz, DMSO-d_6_) δ: 160.04 (C-2), 153.04 (C-8a), 139.10 (C-4), 131.83 (C-7), 128.75 (C-5), 126.04 (C-3), 125.15 (C-6), 119.43 (C-4a), 116.56 (C-8), 103.18 (C-1′), 77.50 (C-4′ or C-3′), 77.03 (C-5′), 74.03 (C-2′), 70.53 (C-4′ or C-3′), 65.24 (CH_2_), 61.53 (C-6′).

1-*O*-[2*H*-chromene-2-one-3-yl-methyl]-β-D-galactopyranoside (**8b**). Yield: 55%. Yellowish sticky solid.

^1^H-NMR (500 MHz, DMSO-d_6_) δ: 8.17 (s, 1H, H-4), 7.68 (d, 1H, *J*_5,6_ = 7.7 Hz, H-5), 7.60 (t, 1H, *J*_7,8_ = 7.9 Hz, H-7), 7.43 (d, 1H, *J*_8,7_ = 8.3 Hz, H-8), 7.38 (t, 1H, *J*_6,5_ = 7.6 Hz, H-6), 5.09 (d, 1H, *J* = 4.7 Hz, OH), 4.77 (d, 1H, *J* = 5.5 Hz, OH), 4.66 (d, 1H, *J* = 15.3 Hz, CH_2_), 4.61 (d, 1H, *J* = 5.6 Hz, OH), 4.48 (d, 1H, *J* = 15.3 Hz, CH_2_), 4.42 (d, 1H, *J* = 4.5 Hz, OH), 4.27 (d, 1H, *J*_1′,2′_ = 7.6 Hz, H-1′), 3.65 (d, 1H, *J*_3′,4′_ = 3.9 Hz, H-4′), 3.52 (tq, 2H, *J*_6′a,6′b_ = 10.7, *J*_6′,5′_ = 5.2 Hz, H-6′a, H-6′b), 3.44 (t, 1H, *J*_2′,3′_ = 4.1 Hz, H-2′), 3.39 (t, 1H, *J*_5′,6′_ = 6.2 Hz, H-5′), 3.31 (s, 1H, H-3′).

^13^C-NMR (126 MHz, DMSO-d_6_) δ: 160.06 (C-2), 153.03 (C-8a), 139.14 (C-4), 131.83 (C-7), 128.61 (C-5), 126.09 (C-3), 125.14 (C-6), 119.43 (C-4a), 116.55 (C-8), 103.81 (C-1′), 75.87 (C-5′), 73.75 (C-3′), 71.11 (C-2′), 68.63 (C-4′), 65.10 (CH_2_), 60.92 (C-6′).

3-*O*-(2*H*-chromene-2-one-3-yl-methyl)-1,2:5,6-di-*O*-isopropylidene-α-D-glucofuranose (**7a**). Yield: 80%. Colorless oil.

^1^H-NMR (500 MHz, CDCl_3_) δ: 7.90 (d, 1H, *J*_4,5_ = 1.7 Hz, H-4), 7.54 (ddd, 1H, *J* = 8.6 Hz, 7.3 Hz, *J*_5,4_ = 1.6 Hz, H-5), 7.52 − 7.49 (m, 1H, H-7), 7.36 (d, 1H, *J*_7,8_ = 8.3 Hz, H-8), 7.31 (td, 1H, *J* = 7.5, 1.1 Hz, H-6), 5.95 (d, 1H, *J*_1′,2′_ = 3.7 Hz, H-1′), 4.75 − 4.70 (m, 1H, H-2′), 4.69 − 4.67 (m, 1H, CH_2_), 4.60 (dd, 1H, *J* = 15, 1.5 Hz, CH_2_), 4.43 (ddd, 1H, *J*_5′,6′a_ = 8.6, *J*_5′,6′b_ = 6.1, *J*_5′,4′_ = 5.1 Hz, H-5′), 4.21 − 4.15 (m, 1H, H-3′), 4.15 − 4.12 (m, 1H, H-6′b), 4.11 (d, 1H, *J*_4′,5′_ = 3.1 Hz, H-4′), 4.07 (dd, 1H, *J*_6′a,6′b_ = 8.6, *J*_6′a,5′_ = 5.1 Hz, H-6′a), 1.53 (s, 3H, CH_3_), 1.43 (s, 3H, CH_3_), 1.39 (s, 3H, CH_3_), 1.35 (s, 3H, CH_3_).

^13^C-NMR (126 MHz, CDCl_3_) δ: 160.36 (C-2), 153.23 (C-8a), 138.81 (C-4), 131.34 (C-5), 127.74 (C-7), 125.42 (C-3), 124.55 (C-6), 119.15 (C(CH_3_)_2_), 116.63 (C-8), 111.99 (C-4a), 109.28 (C(CH_3_)_2_), 105.28 (C-1′), 82.46 (C-2′), 82.14 (C-4′), 81.16 (C-3′), 72.27 (C-5′), 67.60 (C-6′), 66.61 (CH_2_), 26.95 (CH_3_), 26.79 (CH_3_), 26.20 (CH_3_), 25.45 (CH_3_).

6-*O*-(2*H*-chromene-2-one-3-yl-methyl)-1,2:3,4-di-*O*-isopropylidene-α-D-galactopyranose (**8a**). Yield: 98%. Yellowish sticky solid.

^1^H-NMR (500 MHz, CDCl_3_) δ: 7.86 (s, 1H, H-4), 7.50 (dd, 1H, *J*_5,6_ = 4.4, *J*_5,4_ = 2.7 Hz, H-5), 7.49 − 7.47 (m, 1H, H-7), 7.34 − 7.31 (m, 1H, H-8), 7.30 − 7.27 (m, 1H, H-6), 5.57 (d, 1H, *J*_1′,2′_ = 5.0 Hz, H-1′), 4.64 (dd, 1H, *J*_3′,4′_ = 7.9, *J*_3′,2′_ = 2.5 Hz, H-3′), 4.56 (dd, 1H, *J* = 15.3 Hz, CH_2_), 4.51 (dd, 1H, *J* = 15.3 Hz, CH_2_), 4.34 (dd, 1H, *J*_2′,1′_ = 5.0 Hz, *J*_2′,3′_ = 2.4 Hz, H-2′), 4.31 (dd, 1H, *J*_4′,3′_ = 7.9, *J*_4′,5′_ = 1.9 Hz, H-4′), 4.09 (ddd, 1H, *J*_5′,6′a_ = 7.2 Hz, *J*_5′,6′b_ = 5.4 Hz, *J*_5′,4′_ = 2.0 Hz, H-5′), 3.82 (dd, 1H, *J*_6′b,6′a_ = 10.2 Hz, *J*_6′b,5′_ = 5.5 Hz, H-6′b), 3.77 (dd, 1H, *J*_6′a,6′b_ = 10.2 Hz, *J*_6′a,5′_ = 7.0 Hz, H-6′a), 1.56 (s, 3H, CH_3_), 1.43 (s, 3H, CH_3_), 1.35 (s, 3H, CH_3_), 1.34 (s, 3H, CH_3_).

^13^C-NMR (126 MHz, CDCl_3_) δ: 160.43 (C-2), 153.11 (C-8a), 138.52 (C-4), 131.02 (C-5), 127.77 (C-7), 125.96 (C-3), 124.41 (C-6), 119.27 (C(CH_3_)_2_), 116.50 (C-8), 109.37 (C-4a), 108.64 (C(CH_3_)_2_), 96.37 (C-1′), 71.19 (C-4′), 70.69 (C-3′), 70.53 (C-2′), 70.04 (C-6′), 67.76 (CH_2_), 66.73 (C-5′), 26.08 (CH_3_), 25.98 (CH_3_), 24.93 (CH_3_), 24.50 (CH_3_).

### 3.8. General Procedure for Removal of Isopropylidene Protecting Groups

Corresponding di-*O*-isopropylidene-protected glycosyl coumarin (1 mmol) was dissolved in 14 mL per g of reactive in a 1:1 water and trifluoroacetic acid mixture, and reaction mixture was stirred for 24 h at room temperature. Crude products were purified on column chromatography with dichloromethane:methanol (9:1) as eluent; alpha and beta anomers could not be separated by column chromatography [20].

3-*O*-(2*H*-chromene-2-one-3-yl-methyl)-D-glucopyranose (**9a**). Yield: 69%. Yellowish sticky solid.

^1^H-NMR (500 MHz, DMSO-d_6_, α/β = 48:52) δ: 8.02 (d, 1H, *J*_4,5_ = 1.4 Hz, H-4), 7.78 (dd, 1H, *J*_5,6_ = 7.7 Hz, *J*_5,4_ = 1.6 Hz, H-5), 7.60 (ddd, 1 H, *J*_7,8_ = 8.3 Hz, *J*_7,6_ = 7.3 Hz, *J*_7,5_ = 1.6 Hz, H-7), 7.42 (dd, 1H, *J*_8,7_ = 8.3 Hz, *J*_8,6_ = 1.1 Hz, H-8), 7.37 (td, 1H, *J* = 7.4, 1.1 Hz, H-6), 6.58 (d, 0.5H, *J* = 6.6 Hz, OH), 6.21 (dd, 0.5H, *J* = 4.7, 0.9 Hz, OH), 4.97 (t, 0.5H, *J* = 4.1 Hz, H-1′), 4.74 (d, 0.5H, *J* = 4.5 Hz, OH), 4.69 (d, 0.5H, *J* = 5.4 Hz, OH), 4.52 (d, 0.5H, *J* = 5.6 Hz, OH), 4.49 (d, 0.5H, *J* = 4.4 Hz, OH), 4.44 (d, 1H, CH_2_), 4.39 (dd, 1H, CH_2_), 4.31 (d, *J* = 6.9 Hz, OH), 4.28 (dd, 0.5H, *J* = 7.4, 6.5 Hz, β-H-1′), 4.07 (t, *J* = 6.1 Hz, OH), 3.76 − 3.67 (m, 0.5H, α-H-6′), 3.67 − 3.61 (m, 1.5H, α-H-4′, H-3′), 3.60 − 3.55 (m, 1H, β-H-4′, α-H-6′), 3.55 − 3.50 (m, 1H, α-H-2′, β-H-6′), 3.30 (ddd, 1H, *J* = 9.5, 5.4, 3.2 Hz, H-5′), 3.25 (ddd, 0.5H, *J* = 9.6, 7.4, 4.5 Hz, β-H-2′).

^13^C-NMR (126 MHz, DMSO-d_6_, α/β = 48:52) δ: 160.08 (C-2), 153.10 (C-8a), 139.11 (C-4), 131.87 (C-7), 128.83 (C-5), 126.06 (C-3), 125.07 (C-6), 119.39 (C-4a), 116.49 (C-8), 97.89 (β-C-1′), 93.14 (α-C-1′), 73.75 (β-C-5′), 73.38 (α-C-4′ or α-C-3′), 72.44 (β-C-2′), 71.03 (C-6′), 69.86 (α-C-5′), 69.68 (α-C-4′ or α-C-3′), 69.24 (β-C-4′ or β-C-3′), 69.11 (β-C-4′ or β-C-3′), 68.99 (α-C-2′), 67.59 (CH_2_).

6-*O*-(2*H*-chromene-2-one-3-yl-methyl)-D-galactopyranose (**10a**). Yield: 78%. White sticky solid

^1^H-NMR (500 MHz, DMSO-d_6_, α/β = 44:56) δ: 8.02 (d, 1H, *J* = 1.4 Hz, H-4), 7.80 − 7.68 (m, 1H, H-5), 7.60 (t, 1H, *J* = 7.9 Hz, H-7), 7.45 − 7.33 (d, 1H, *J*_8,7_ = 8.2 Hz, H-8), 7.27 (m, 1H, H-6), 6.58 (d, *J* = 6.5 Hz, β-OH), 6.20 (d, *J* = 4.7 Hz, α-OH), 5.48 (d, 0.5H, *J*_1′-2′_ = 5.0 Hz, α-H-1′), 4.96 (d, *J* = 4.0 Hz, OH), 4.73 (d, *J* = 4.4 Hz, OH), 4.68 (d, *J* = 5.3 Hz, OH), 4.60 (dd, *J* = 8.4, 2.6 Hz, OH), 4.51 (d, *J* = 5.6 Hz, OH), 4.48 (d, *J* = 4.2 Hz, OH), 4.44 − 4.37 (m, 2H, CH_2_), 4.35 (dd, *J* = 5.2, 2.1 Hz, OH), 4.33 − 4.25 (m, 0.5H, *J*_1′,2′_ = 4.2 Hz, β-H-1′), 3.77 − 3.48 (m, 4H, H-6′a,H-6′b, H-3′, H-4′), 3.33 − 3.19 (m, 2H, H-5′, H-2′).

^13^C-NMR (126 MHz, DMSO-d_6_, α/β = 44:56) δ: 160.09 (C-2), 153.14 (C-8a), 139.09 (C-4), 131.89 (C-7), 129.76 (C-5), 128.80 (C-3), 126.08 (C-6), 119.39 (C-4a), 116.50 (C-8), 96.12 (C-1′), 73.75 (β-C-5), 73.37 (α-C-4′ or α-C-3′), 72.44 (C-2′), 70.97 (C-6′), 69.83 (α-C-5′), 69.68 (α-C-4′ or α-C-3′), 69.24 (β-C-4′ or β-C-3′), 69.11 (β-C-4′ or β-C-3′), 68.99 (α-C-2′), 67.59 (CH_2_).

### 3.9. Inhibition of Carbonic Anhydrase Isozymes

An Applied Photophysics stopped-flow instrument has been used for assaying the CA catalyzed CO_2_ hydration activity as reported by Khalifah [23]. Phenol red (at a concentration of 0.02 mM) has been used as the indicator, working at the absorbance maximum of 557 nm, with 20 mM Hepes (pH 7.5) as buffer, and 20 mM Na_2_SO_4_ (for maintaining constant the ionic strength), following the initial rates of the CA-catalyzed CO_2_ hydration reaction for a period of 10–100 s. The CO_2_ concentrations ranged from 1.7 to 17 mM for the determination of the kinetic parameters and inhibition constants. For each inhibitor, at least six traces of the initial 5–10% of the reaction have been used for determining the initial velocity. The uncatalyzed rates were determined in the same manner and subtracted from the total observed rates. Stock solutions of inhibitor (0.1 mM) were prepared in distilled–deionized water, and dilutions up to 0.01 nM were performed thereafter with distilled–deionized water. Inhibitor and enzyme solutions were preincubated together for 15 min at room temperature prior to assay in order to allow for the formation of the E-I complex. The inhibition constants were obtained by non-linear least-squares methods using PRISM 3, and the Cheng–Prusoff equation [24] as reported in earlier work from our groups [25,26,27], and represent the mean from at least three different determinations. All *h*CAs used in the experiments were recombinant proteins obtained in one of our laboratories as described in earlier work [25,26,27].

## 4. Computational Methodology

### 4.1. Sequence Alignment and System Preparation

Multiple sequence alignment of *h*CA isoforms I, II, IX, and XII was performed using Clustal Omega (accessed 1 November 2025) [28] through the UniProt interface. Alignment visualization was conducted with SeaView Version 5.0.5 [29]. Residue numbering throughout this study follows the UniProt canonical sequences for *h*CA I (P00915), *h*CA II (P00918), *h*CA IX (Q16790), and *h*CA XII (O43570).

The X-ray crystal structures used for docking and molecular dynamics (MD) simulations were retrieved from the Protein Data Bank (PDB) with the following accessions: 2FW4 (*h*CA I), 3F8E (*h*CA II), 6Y74 (*h*CA IX), and 9FN7 (*h*CA XII).

### 4.2. Molecular Docking

**10a** anomers were prepared using OMEGA [30] to generate conformational ensembles in POSE mode with default settings. Receptor sites were defined and prepared using the Make Receptor utility, centered on co-crystallized ligands. Molecular docking was performed using FRED [31], employing the Chemgauss4 scoring function to identify the most favorable binding poses for both α and β anomers within each *h*CA isoform. OMEGA, Make Receptor, and FRED are included in OpenEye Scientific Software suite version 2025.2 [32].

### 4.3. Molecular Dynamics Simulations

MD simulations were carried out using NAMD 2.14 [33]. The protein was modeled using the AMBER ff14SB force field [34], while the ligands were parameterized with the General Amber Force Field (GAFF) [35]. Partial atomic charges for the ligands were derived using the RESP method [36] calculated at the HF/6-31G* level with Gaussian 03 [37]. The catalytic center (Zn^2+^ and coordinating residues) was treated using the Zinc Amber Force Field (ZAFF) [38] to ensure proper coordination geometry. The Zn(II) ion was modeled in a tetrahedral coordination environment ZN6, consisting of three conserved histidine residues and one water molecule. The complexes were solvated in a cubic box of TIP3P water molecules extending 15 Å from the solute atoms and neutralized with Cl^−^. A cutoff of 12.0 Å (switching at 10.0 Å) was applied. The simulation protocol consisted of four successive minimization stages: (i) hydrogen atoms only, (ii) water molecules and ions, (iii) side chains with fixed backbone, (iv) and total system minimization. Systems were heated from 20 K to 310 K over 300 ps using Langevin dynamics. Equilibration was performed in the NPT ensemble at 310 K and 1 bar using the Langevin piston method for 2 ns and 1 fs timestep. Production runs of 100 ns were executed with a 2 fs timestep using the SHAKE algorithm to constrain bonds involving hydrogen.

### 4.4. Binding Free Energy and Interaction Fingerprints

Binding free energies were estimated using the Molecular Mechanics Generalized Born Surface Area (MM-GBSA) method via MMPBSA.py [39]. Calculations were performed on the last 50 ns, every 5000 steps, from the production trajectories using the igb = 5 model with a salt concentration of 0.1 M. Per-residue energy decomposition was conducted with idecomp = 3 option to identify individual amino acid contributions to the binding affinity. The entropy term was neglected in the final ΔG_bind_ estimation based on the structural similarity of the 10a anomers, assuming that their entropic contributions cancel out in comparative analyses.

Dynamic protein–ligand interaction fingerprints were computed using ProLIF [40]. Interactions including hydrogen bonds, van der Waals contacts, and hydrophobic effects were monitored across the trajectories to quantify the occupancy and stability of the observed binding modes.

## 5. Conclusions

In summary, an efficient synthetic route to glycosylated coumarins was developed through a copper-catalyzed multicomponent reaction starting from propargyl glycosides derived from natural carbohydrates. This methodology enables the rapid generation of structurally diverse glycosyl coumarins bearing an iminocoumarin core.

Biological evaluation revealed that the synthesized compounds exhibit a clear selectivity profile toward tumor-associated carbonic anhydrase isoforms *h*CA IX and *h*CA XII while showing negligible inhibition of the cytosolic isoforms *h*CA I and *h*CA II. Among the compounds tested, **10a** showed the highest inhibitory activity toward both tumor-associated isoforms.

Structure–activity relationship analysis suggested that the presence of deprotected glycosyl moieties enhances inhibitory potency, likely due to increased polarity and improved hydrogen-bonding interactions at the enzyme surface.

Computational studies combining molecular docking, molecular dynamics simulations, and MM-GBSA binding free energy calculations provided structural insight into the observed selectivity. Between the anomers of compound **10a**, the β-anomer showed the most favorable binding energies and established key stabilizing interactions with conserved catalytic residues and isoform-specific hotspots in *h*CA IX and XII.

Overall, this study demonstrates that glycosyl coumarins represent a valuable starting point for the design and optimization of selective inhibitors targeting tumor-associated carbonic anhydrases. Further structural optimization of this scaffold may lead to compounds with improved potency and potential therapeutic applications.

## Figures and Tables

**Table 1:** Inhibition of *h*CA isoforms I, II, IX, and XII with glycosyl coumarins and acetazolamide (AAZ; 5-acetamido-1,3,4-thiadiazole-2-sulfonamide) as standard by a stopped-flow CO_2_ hydrase assay.

	Ki (μM) ^a^			
	CA I	CA II	CA IX	CA XII
**7a**	>100	>100	33.2 ± 1.7	29.4 ± 0.9
**7b**	>100	>100	41.8 ± 2.5	31.0 ± 2.0
**8a**	>100	>100	36.9 ± 2.4	35.6 ± 1.8
**8b**	>100	>100	38.5 ± 3.1	26.7 ± 1.5
**9a**	>100	>100	22.3 ± 1.9	24.1 ± 0.8
**10a**	>100	>100	12.9 ± 0.8	19.6 ± 1.1
**AAZ**	0.25	0.012	0.025 ± 0.001	0.0057 ± 0.0002

^a^ Mean ± standard error, from three different replicates.

**Table 2:** Calculated MM-GBSA binding free energies (ΔG_bind_) for the complexes of *h*CA I, II, IX, and XII with the α and β anomers of the ligand **10a**. These calculations were performed using the MMPBSA.py module, as implemented in AmberTools25.

Complex	MM-GBSA Binding Energy (kcal mol^−1^)
hCA I (2FW4)/α-**10a**	−34.1 ± 4.3
hCA I (2FW4)/β-**10a**	−32.6 ± 4.7
hCA II (3F8E)/α-**10a**	−39.1 ± 7.9
hCA II (3F8E)/β-**10a**	−40.0 ± 4.9
hCA IX (6Y74)/α-**10a**	−43.3 ± 5.3
hCA IX (6Y74)/β-**10a**	−47.1 ± 3.8
hCA XII (9FN7)/α-**10a**	−38.8 ± 4.7
hCA XII (9FN7)/β-**10a**	−49.5 ± 5.7

## Data Availability

The original contributions presented in this study are included in the article/Supplementary Materials. Further inquiries can be directed to the corresponding authors.

## Supplementary Materials

The following supporting information can be downloaded at: https://www.mdpi.com/article/10.3390/ijms27083659/s1.
