# Supplementary material for: Glycosyl Coumarins as Selective Inhibitors of Tumor-Associated Carbonic Anhydrase IX and XII: Synthesis, Structure–Activity Relationships, and Molecular Modeling
Source: Int J Mol Sci. 2026 Apr 20;27(8):3659. doi: 10.3390/ijms27083659 (PMC13116586; doi:10.3390/ijms27083659)
Supplement: Supplementary file 1 [file ijms-27-03659-s001.zip › ijms-4250527-supplementary/final_supplementary_information.pdf]

Glycosyl Coumarins as Selective of Tumor-Associated Carbonic Anhydrase IX and XII Inhibitors: Synthesis, Structure–Activity Relationships, and Molecular Modeling

Macarena Le Pors, #[a] Ignacio Aznar,#[a] Simone Giovannuzzi,[c] Claudiu T. Supuran[c] Martín J. Lavecchia, *[b] and Pedro A. Colinas*[a]

# Both authors contributed equally to this report

[a]CEDECOR (UNLP-CICBA), CONICET, Departamento de Química, Facultad de Ciencias Exactas, Universidad Nacional de La Plata, 47 y 115, 1900 La Plata, Argentina
E-mail: pcolinas@quimica.unlp.edu.ar
[b] CEQUINOR (UNLP-CONICET, CCT-La Plata, associated with CIC), Departamento de Química, Facultad de Ciencias Exactas, Universidad Nacional de La Plata Blvd. 120 1465, La Plata, Argentina
[c] Università degli Studi di Firenze, NEUROFARBA Department, Section of Pharmaceutical Chemistry, Via Ugo Schiff 6, 50019 Sesto Fiorentino (Florence), Italy

TABLE OF CONTENTS

Figure S1. RMSD trajectory analysis for the *h*CA I complexes S3

Figure S2. RMSD trajectory analysis for the *h*CA II complexes S4

Figure S3. RMSD trajectory analysis for the *h*CA IX complexes S5

Figure S4. RMSD trajectory analysis for the *h*CA XII complexes S6

Figure S5. Representative binding modes of the **10a** anomers within the *h*CA I and *h*CA XII active sites S7

Figure S6. Multiple sequence alignment for the *h*CAs S8

Figure S7. Structural reorganization of the α-**10a** anomer within the *h*CA I active site S9

Figure S8. ^{1}H NMR spectra of 3-*O*-(2*H*-chromene-2-one-3-yl-methyl)-1,2:5,6-di-*O*-isopropylidine-α-D-glucofuranose (**7a**) S10

Figure S9. ^{13}C NMR spectra of 3-*O*-(2*H*-chromene-2-one-3-yl-methyl)-1,2:5,6-di-*O*-isopropylidine-α-D-glucofuranose (**7a**) S11

Figure S10. ^{1}H NMR spectra of 1-*O*-[2*H*-chromene-2-one-3-yl-methyl]-β-D-glucopyranoside (**7b**) S12

Figure S11. ^{13}C NMR spectra of 1-*O*-[2*H*-chromene-2-one-3-yl-methyl]-β-D-glucopyranoside (**7b**) S13

Figure S12. ^{1}H NMR spectra of 6-*O*-(2*H*-chromene-2-one-3-yl-methyl)-1,2:3,4-di-*O*-isopropylidene-α-D-galactopyranose (**8a**) S14

Figure S13. ^{13}C NMR spectra of 6-*O*-(2*H*-chromene-2-one-3-yl-methyl)-1,2:3,4-di-*O*-isopropylidene-α-D-galactopyranose (**8a**) S15

Figure S14. ^{1}H NMR spectra of 1-*O*-[2*H*-chromene-2-one-3-yl-methyl]-β-D-galactopyranoside (**8b**) S16

Figure S15. ^{13}C NMR spectra of 1-*O*-[2*H*-chromene-2-one-3-yl-methyl]-β-D-galactopyranoside (**8b**) S17

Figure S16. ^{1}H NMR spectra of 3-*O*-(2*H*-chromene-2-one-3-yl-methyl)-D-glucopyranose (**9a**) S18

Figure S17. ^{13}C NMR spectra of 3-*O*-(2*H*-chromene-2-one-3-yl-methyl)-D-glucopyranose (**9a**) S19

Figure S18. ^{1}H NMR spectra of 6-*O*-(2*H*-chromene-2-one-3-yl-methyl)-D-galactopyranose (**10a**) S20

Figure S19. ^{13}C NMR spectra of 6-*O*-(2*H*-chromene-2-one-3-yl-methyl)-D-galactopyranose (**10a**) S21

Table S1a. Protein–ligand interaction fingerprints for the *h*CA I–α-**10a** complex S22

Table S1b. Protein–ligand interaction fingerprints for the *h*CA I–β-**10a** complex S23

Table S2a. Protein–ligand interaction fingerprints for the *h*CA II–α-**10a** complex S24

Table S2b. Protein–ligand interaction fingerprints for the *h*CA II–β-**10a** complex S25

Table S3a. Protein–ligand interaction fingerprints for the *h*CA IX–α-**10a** complex S26

Table S3b. Protein–ligand interaction fingerprints for the *h*CA IX–β-**10a** complex S27

Table S4a. Protein–ligand interaction fingerprints for the *h*CA XII–α-**10a** complex S28

Table S4b. Protein–ligand interaction fingerprints for the *h*CA XII–β-**10a** complex S29

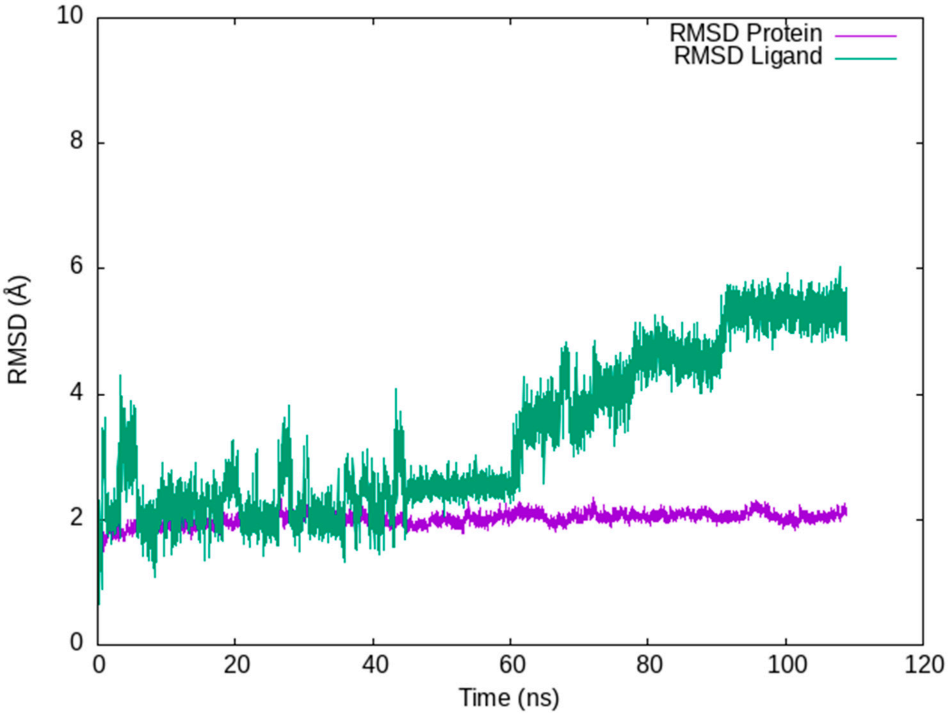

(a) *h*CA I (2FW4) / α-**10a**

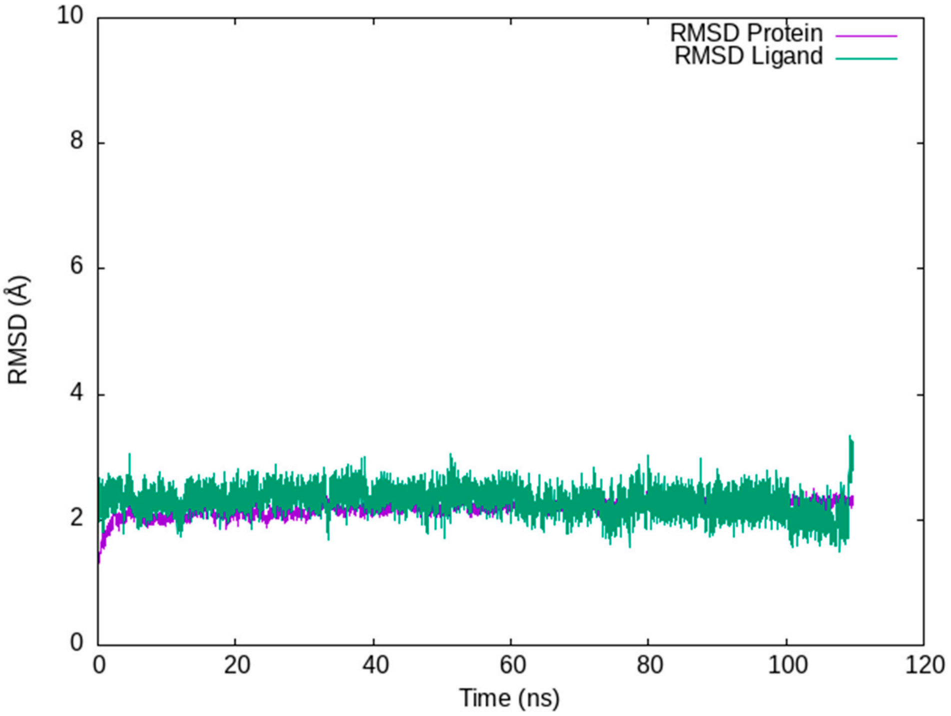

(b) *h*CA I (2FW4) / β-**10a**

Figure S1. RMSD trajectory analysis for the *h*CA I complexes. Time-dependent evolution of the Root Mean Square Deviation (RMSD). Plots were generated with CPPTraj and Gnuplot.

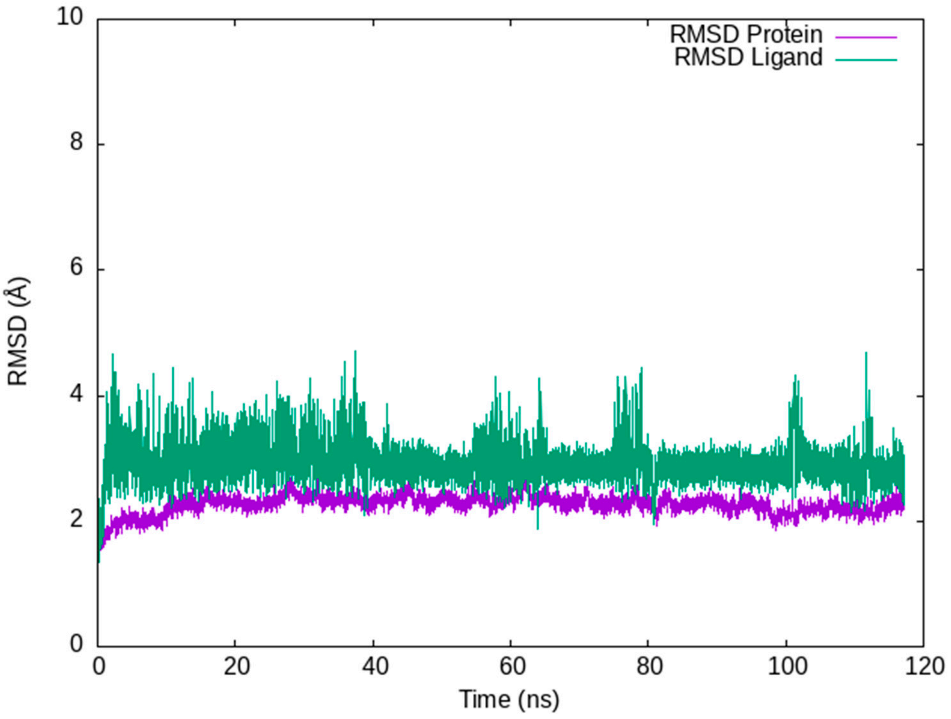

(a) *h*CA II (3F8E) / α-**10a**

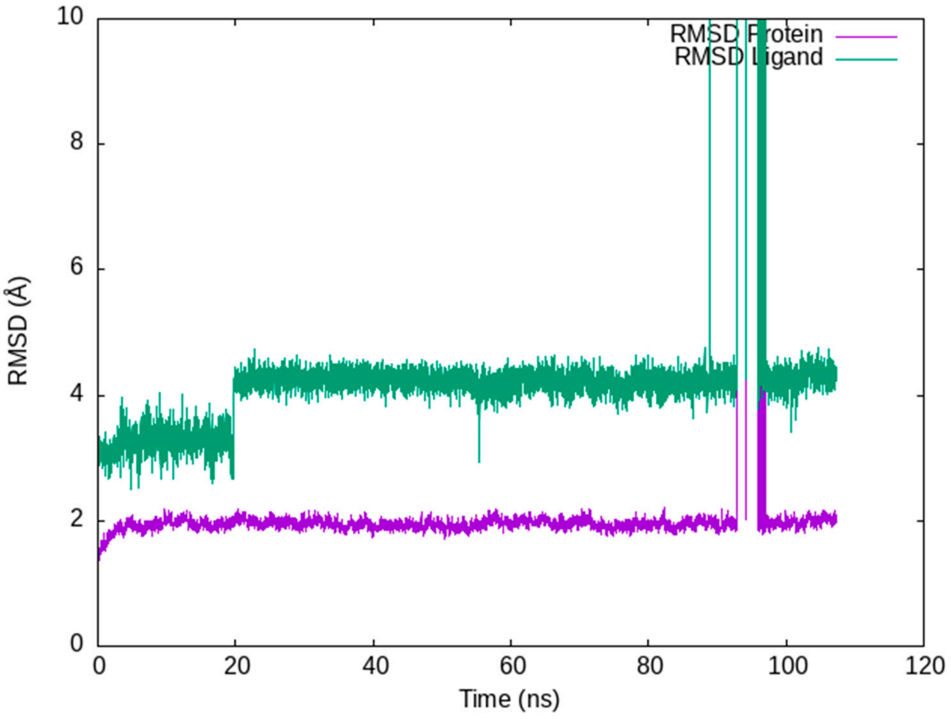

(b) *h*CA II (3F8E) / β-**10a**

Figure S2. RMSD trajectory analysis for the *h*CA II complexes. Time-dependent evolution of the Root Mean Square Deviation (RMSD). Plots were generated with CPPTraj and Gnuplot. *Although some discontinuities are visible in the profiles (e.g., around 95 ns), these were identified as periodic boundary condition (PBC) artifacts and do not impact the subsequent MM-GBSA energy calculations.

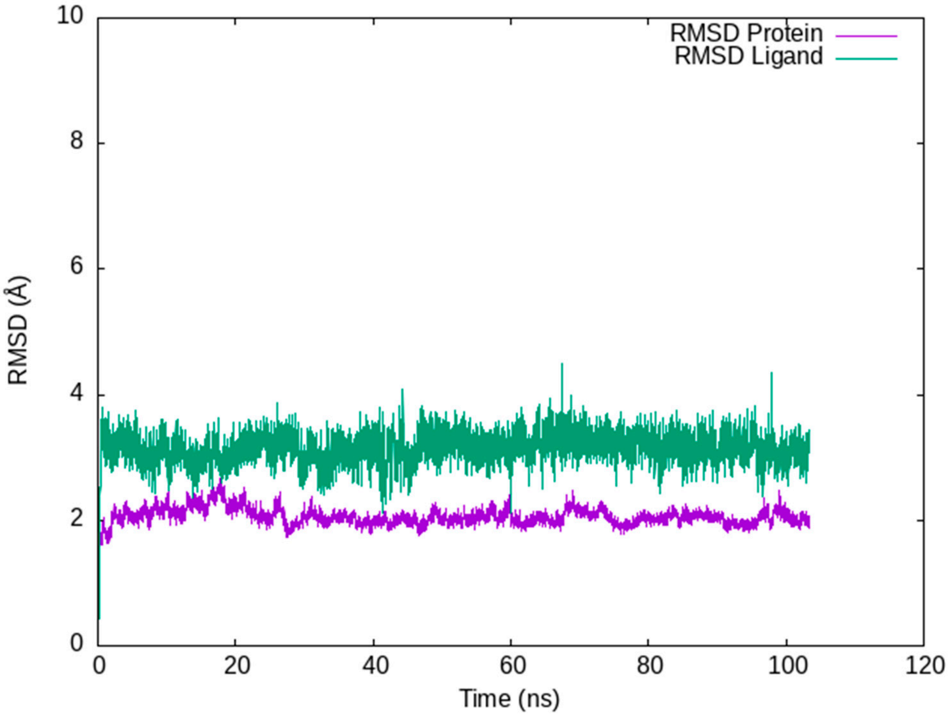

(a) *h*CA IX (6Y74) / α-**10a**

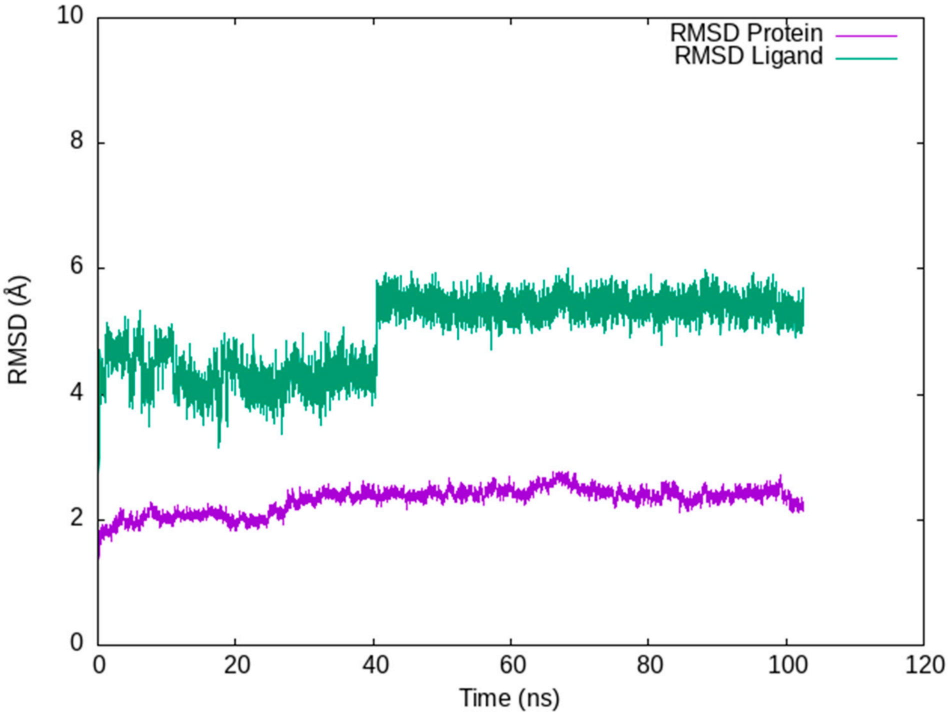

(b) *h*CA IX (6Y74) / β-**10a**

Figure S3. RMSD trajectory analysis for the *h*CA IX complexes. Time-dependent evolution of the Root Mean Square Deviation (RMSD). Plots were generated with CPPTraj and Gnuplot.

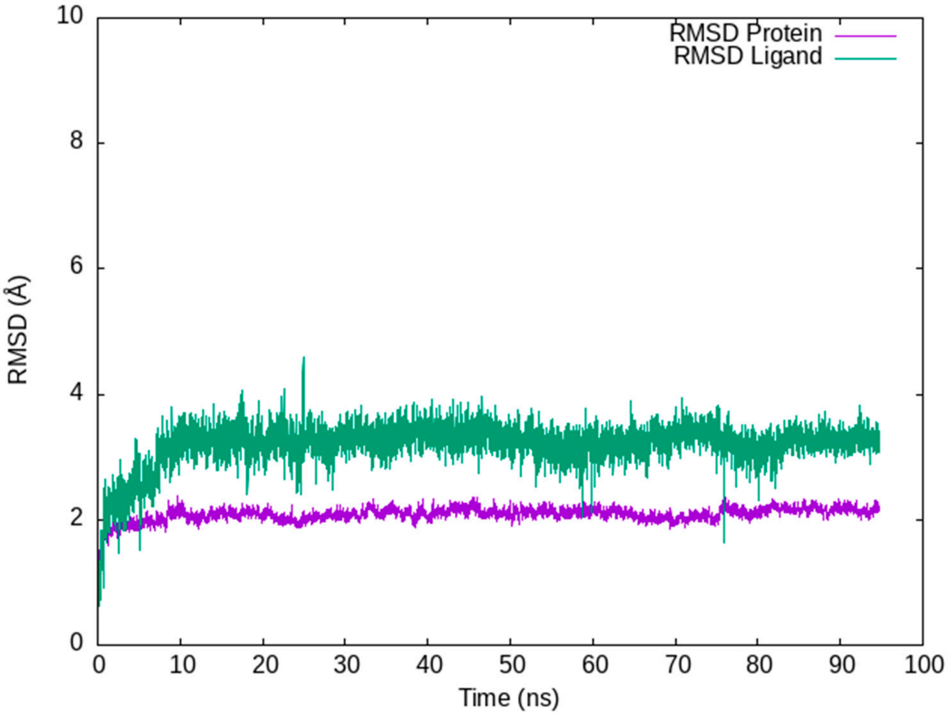

(a) *h*CA XII (9FN7) / α-**10a**

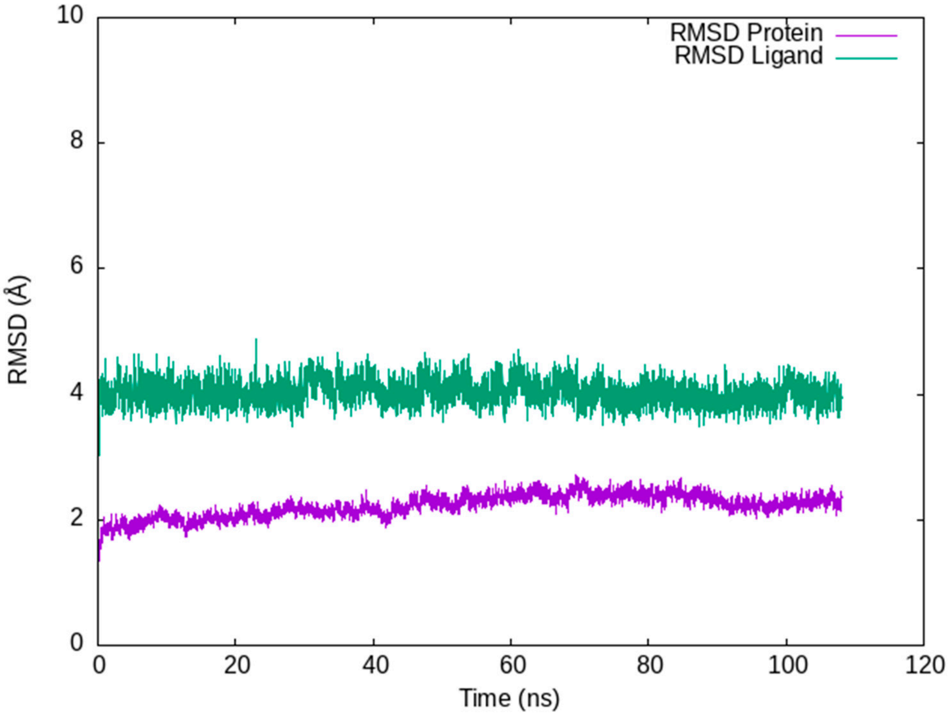

(b) *h*CA XII (9FN7) / β-**10a**

Figure S4. RMSD trajectory analysis for the *h*CA XII complexes. Time-dependent evolution of the Root Mean Square Deviation (RMSD). Plots were generated with CPPTraj and Gnuplot.

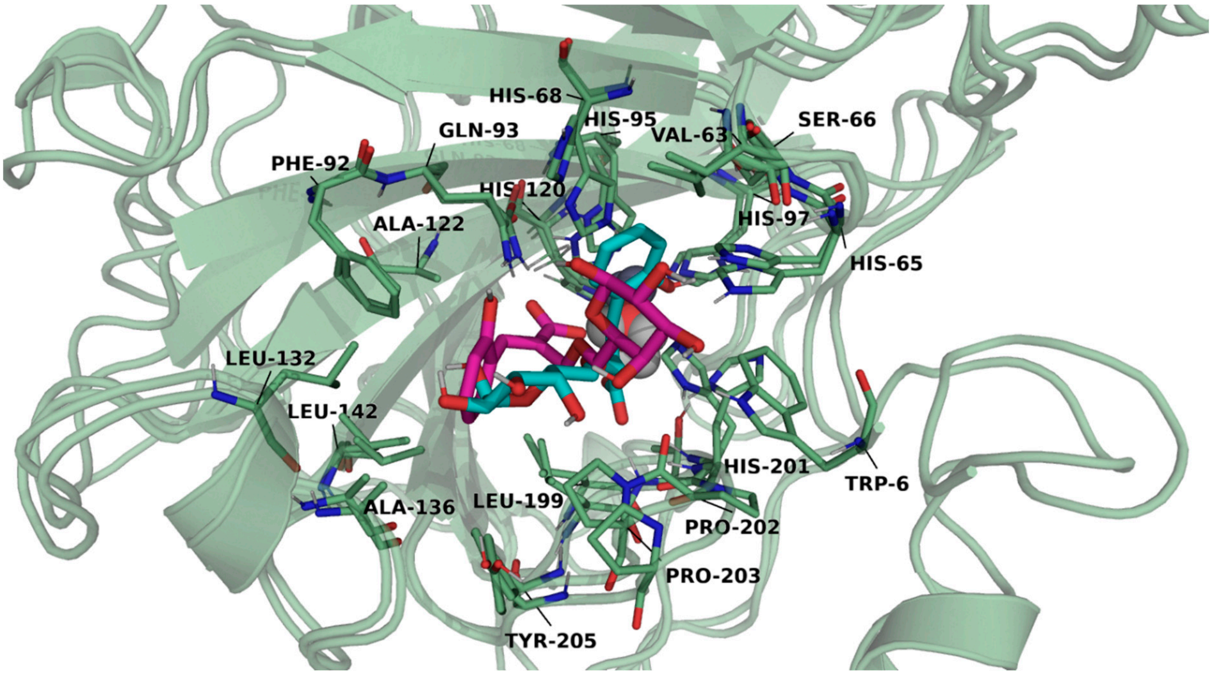

(a)

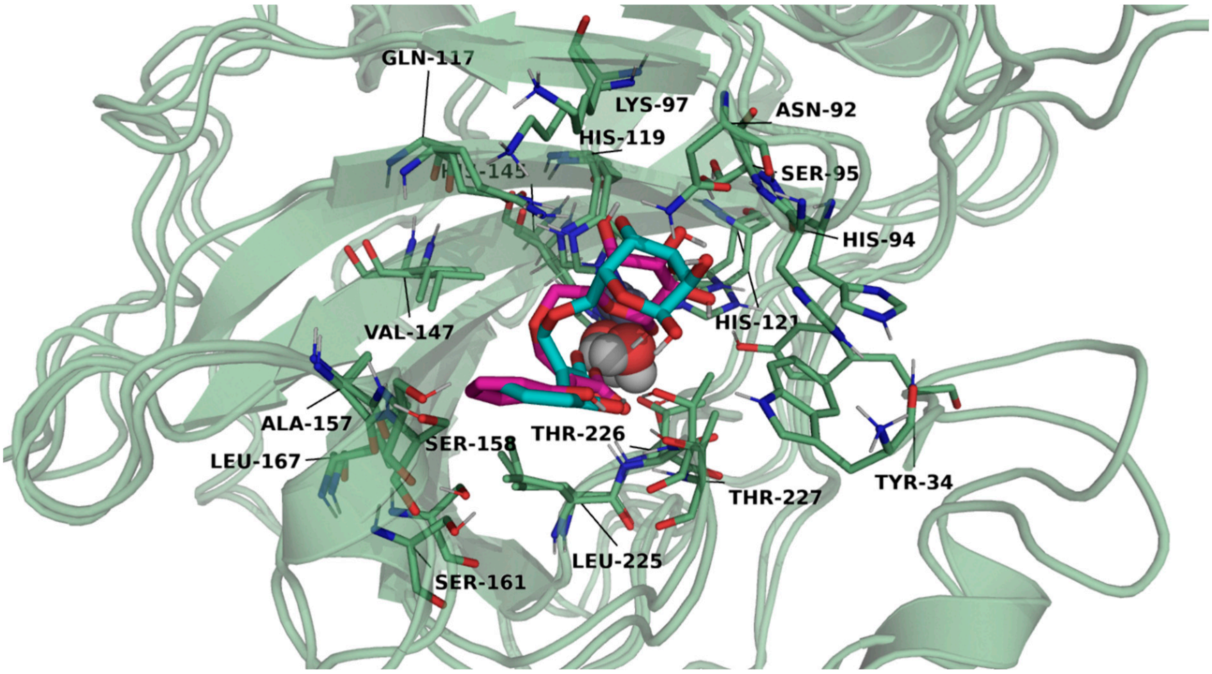

(b)

Figure S5. Representative binding modes of the **10a** anomers within the **(a)** *h*CA I and **(b)** *h*CA XII active sites. Structural superposition of the last frame extracted from the MD trajectories. The α-**10a** is shown in cyan, while the β-**10a** is represented in pink. The protein backbone and key catalytic residues are shown as ribbons/sticks, and the catalytic Zinc ion is depicted as a gray sphere.

```
sp|Q16790|CAH9_  MAPLCPSPWL PLLIPAPAPG LTVQLLLSLL LLVPVHPQRL PRMQEDSPLG GGSSGEDDPL
sp|O43570|CAH12  ---------- ---------- ---------- ---------- ---------- ----------
sp|P00918|CAH2_  ---------- ---------- ---------- ---------- ---------- ----------
sp|P00915|CAH1_  ---------- ---------- ---------- ---------- ---------- ----------

               61
sp|Q16790|CAH9_  GEEDLPSEED SPREEDPPGE EDLPGEEDLP GEEDLPEVKP KSEEEGSLKL EDLPTVEAPG
sp|O43570|CAH12  ---------- ---------- ---------- --------MP RRSLHAAAVL LLVILKEQPS
sp|P00918|CAH2_  ---------- ---------- ---------- ---------- ---------- ----------
sp|P00915|CAH1_  ---------- ---------- ---------- ---------- ---------- ----------

              121
sp|Q16790|CAH9_  DPQEPQNNAH RDKEGDDQSH WRYG---GDP PWPRVSPACA GRFQSPVDIR PQLAAFCPAL
sp|O43570|CAH12  ---------- -SPAPVNGSK WTYFGPDGEN SWSKKYPSCG GLLQSPIDLH SDILQYDASL
sp|P00918|CAH2_  ---------- ------MSHH WGYGKHNGPE HWHKDFPIAK GERQSPVDID THTAKYDPSL
sp|P00915|CAH1_  ---------- -----MASPD WGYDDKNGPE QWSKLYPIAN GNNQSPVDIK TSETKHDTSL

              181
sp|Q16790|CAH9_  RPLELLGFQL PPLPELRLRN NGHSVQLTLP PGLEMAL--- -GPGREYRAL QLHLHWGAA-
sp|O43570|CAH12  TPLEFQGYNL SANKQFLLTN NGHSVKLNLP SDM-----HI QGLQSRYSAT QLHLHWGNPN
sp|P00918|CAH2_  KPLSVS-YDQ A--TSLRILN NGHAFNVEFD DSQDKAVLKG GPLDGTYRLI QFHFHWGSL-
sp|P00915|CAH1_  KPISVS-YNP A--TAKEIIN VGHSFHVNFE DNDNRSVLKG GPFSDSYRLF QFHFHWGST-

              241
sp|Q16790|CAH9_  GRPGSEHTVE GHRFPAEIHV VHLST-AFAR VDEALGRPGG LAVLAAFLEE GPEENSAYEQ
sp|O43570|CAH12  DPHGSEHTVS GQHFAAELHI VHYNSDLYPD ASTASNKSEG LAVLAVLIEM GS-FNPSYDK
sp|P00918|CAH2_  DGQGSEHTVD KKKYAAELHL VHWNT-KYGD FGKAVQQPDG LAVLGIFLKV GS-AKPGLQK
sp|P00915|CAH1_  NEHGSEHTVD GVKYSAELHV AHWNSAKYSS LAEAASKADG LAVIGVLMKV GE-ANPKLQK

              301
sp|Q16790|CAH9_  LLSRLEEIAE EGSETQVPGL DISALLPSDF SRYFQYEGSL TTPPCAQGVI WTVFNQTVML
sp|O43570|CAH12  IFSHLQHVKY KGQEAFVPGF NIEELLPERT AEYYRYRGSL TTPPCNPTVL WTVFRNPVQI
sp|P00918|CAH2_  VVDVLDSIKT KGKSADFTNF DPRGLLPES- LDYWTYPGSL TTPPLLECVT WIVLKEPISV
sp|P00915|CAH1_  VLDALQAIKT KGKRAPFTNF DPSTLLPSS- LDFWTYPGSL THPPLYESVT WIICKESISV

              361
sp|Q16790|CAH9_  SAKQLHTLSD TLWG-----P GDSRLQLNFR ATQPLNGRVI EASFPAGVDS SPRAAEPVQL
sp|O43570|CAH12  SQEQLLALET ALYCTHMDDP SPREMINNFR QVQKFDERLV YTSFSQV--- ----------
sp|P00918|CAH2_  SSEQVLKFRK LNFNGEG--E PEELMVDNWR PAQPLKNRQI KASFK----- ----------
sp|P00915|CAH1_  SSEQLAQFRS LLSNVEG--D NAVPMQHNNR PTQPLKGRTV RASF------ ----------

              421
sp|Q16790|CAH9_  NSCLAAGDIL ALVFGLLF-- --A---VTSV AFLVQMRRQH RRGTKGGVSY RPAEVAETGA
sp|O43570|CAH12  QVCTAAGLSL GIILSLALAG ILGICIVVVV SIWLFRRKSI KKGDNKGVIY KPATKMETEA
sp|P00918|CAH2_  ---------- ---------- ---------- ---------- ---------- ----------
sp|P00915|CAH1_  ---------- ---------- ---------- ---------- ---------- ----------

              481
sp|Q16790|CAH9_  --
sp|O43570|CAH12  HA
sp|P00918|CAH2_  --
sp|P00915|CAH1_  --
```

Figure S6. Multiple sequence alignment for the *h*CAs using CLUSTAL W (1.83) and image generated with Seaview 5.0.5.

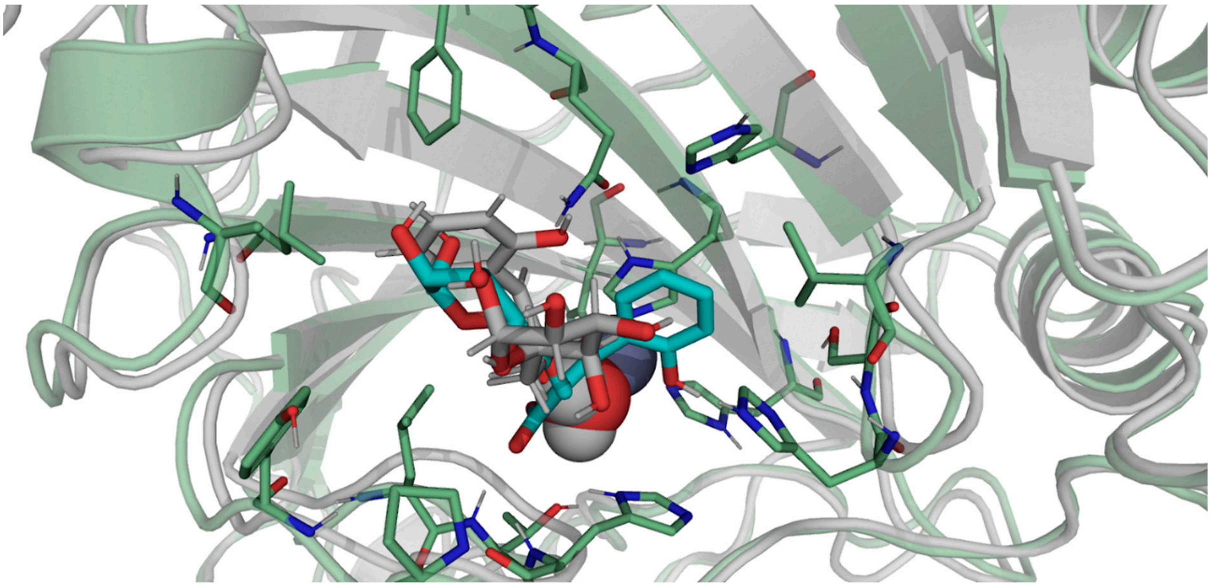

Figure S7. Structural reorganization of the α-**10a** anomer within the *h*CA I active site. Overlay comparing the initial docking pose (gray) and the final snapshot extracted from the molecular dynamics (MD) production trajectory (cyan). The ligand exhibits a significant rotation during the simulation to reach its equilibrated binding mode. The protein backbone is shown in light green ribbons, with key active site residues and the catalytic Zinc ion (sphere) highlighted. Image generated with PyMOL.

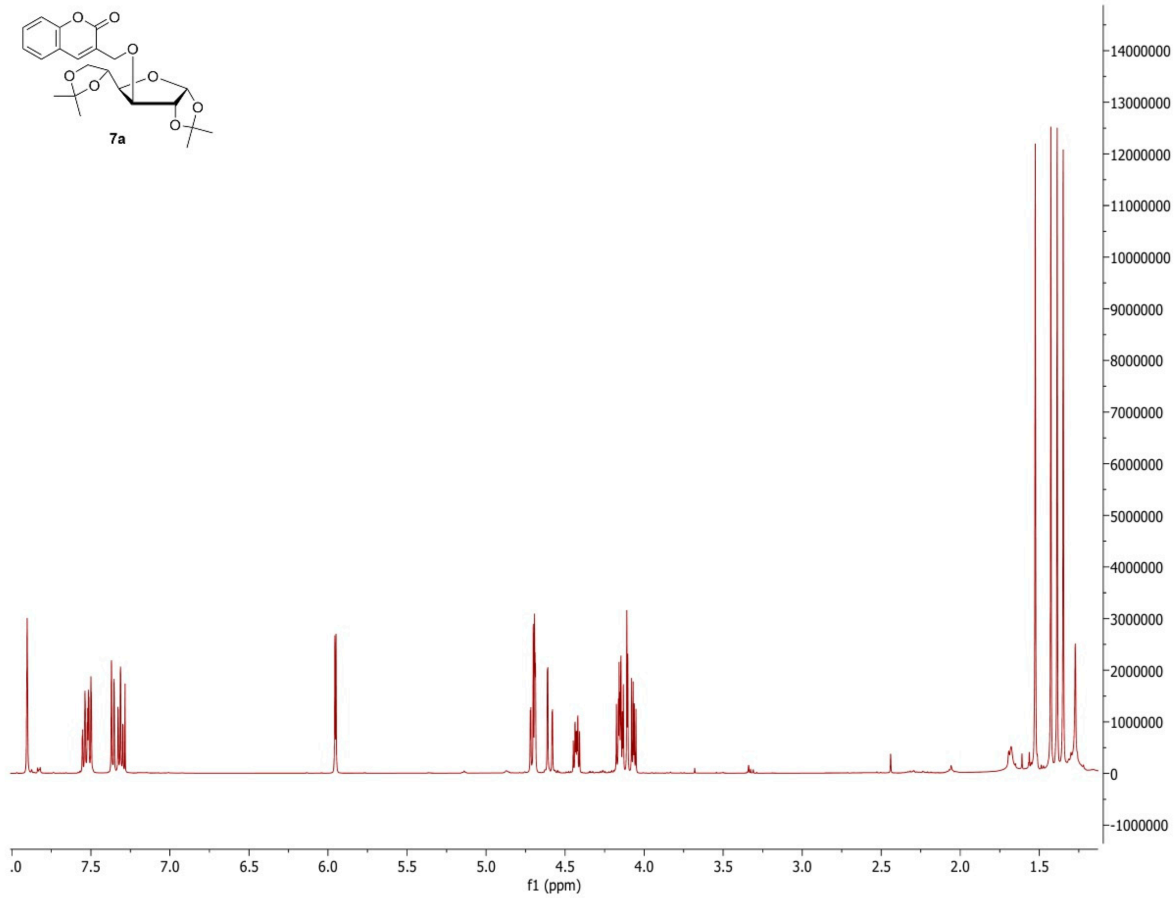

Figure S8. ^{1}H NMR spectra of 3-*O*-(2*H*-chromene-2-one-3-yl-methyl)-1,2:5,6-di-*O*-isopropylidine-α-D-glucofuranose (**7a**).

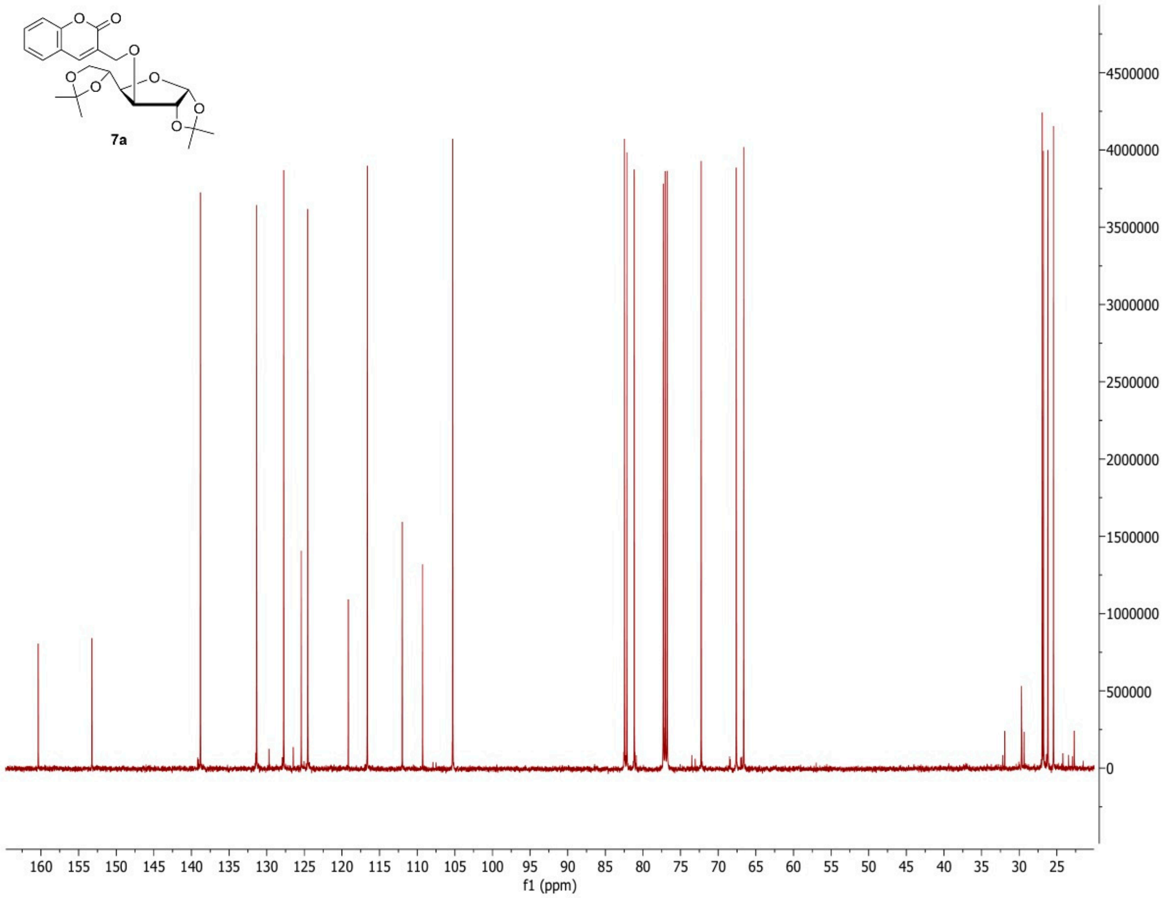

Figure S9. ^{13}C NMR spectra of 3-*O*-(2*H*-chromene-2-one-3-yl-methyl)-1,2:5,6-di-*O*-isopropylidine-α-D-glucofuranose (**7a**).

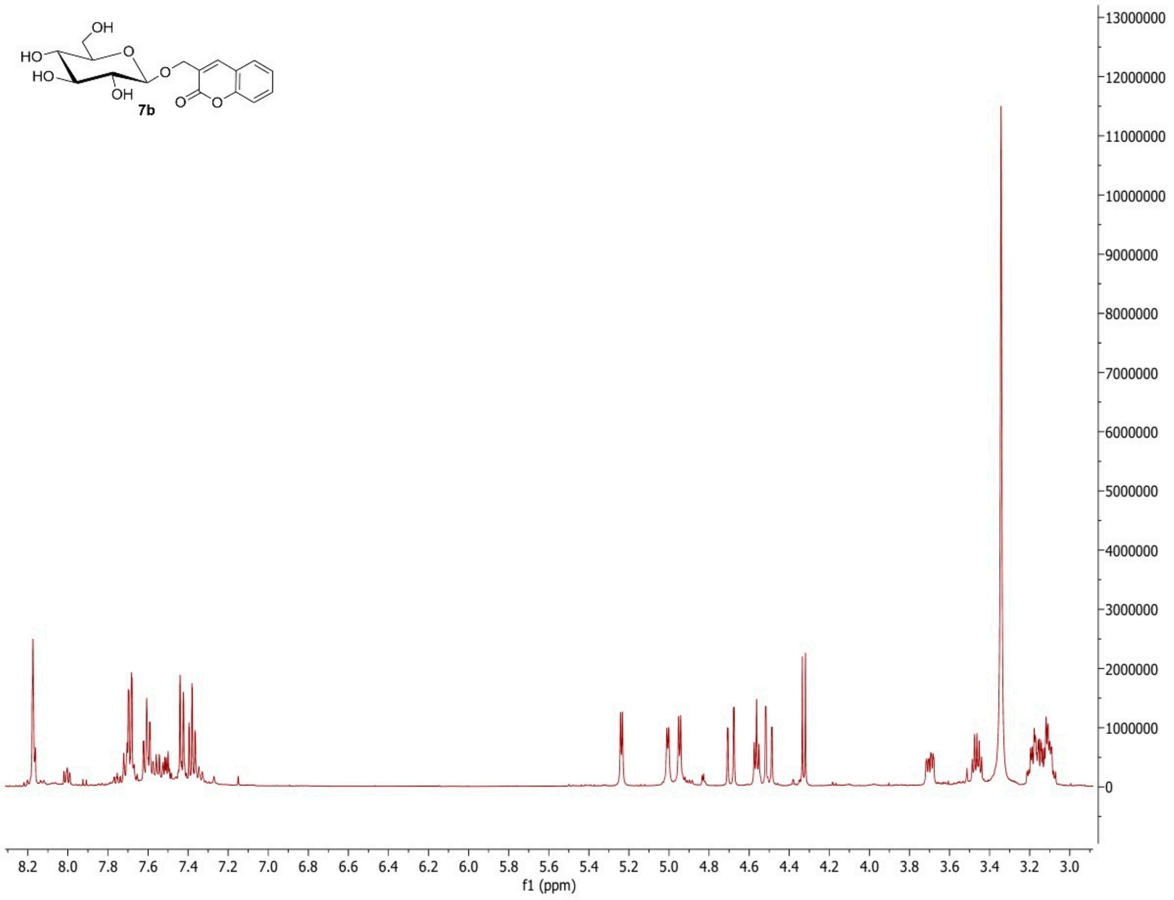

Figure S10. ^{1}H NMR spectra of 1-*O*-[2*H*-chromene-2-one-3-yl-methyl]-β-D-glucopyranoside (**7b**).

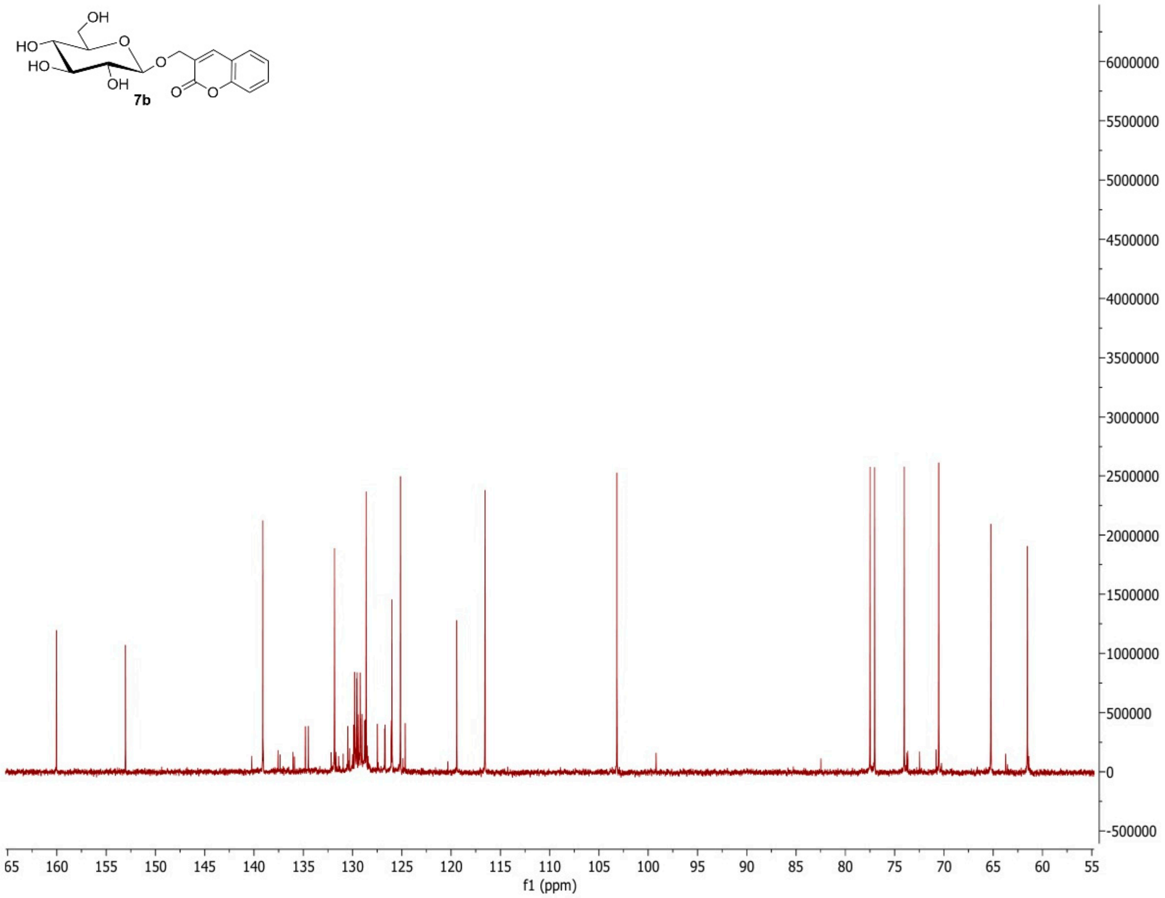

Figure S11. ^{13}C NMR spectra of 1-*O*-[2*H*-chromene-2-one-3-yl-methyl]-β-D-glucopyranoside (**7b**).

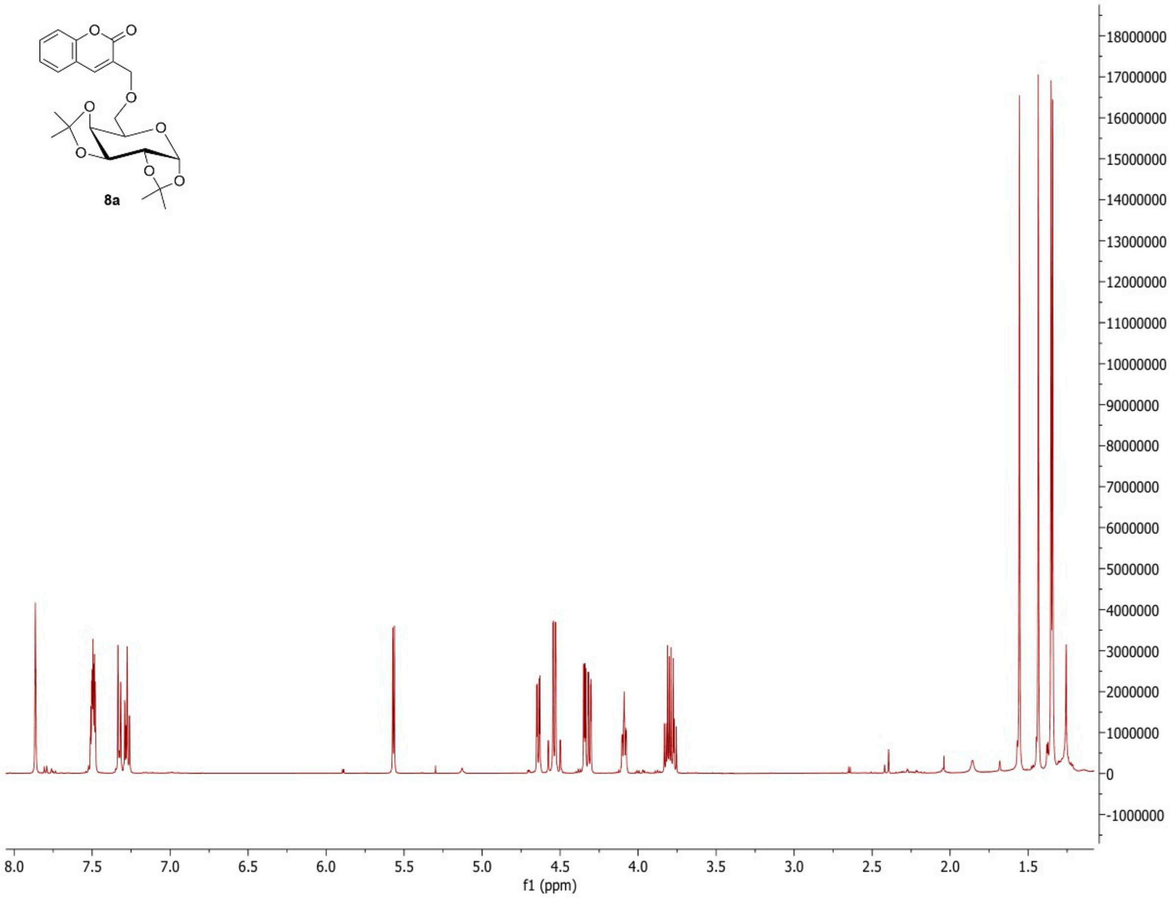

Figure S12. ^{1}H NMR spectra of 6-*O*-(2*H*-chromene-2-one-3-yl-methyl)-1,2:3,4-di-*O*-isopropylidene-α-D-galactopyranose (**8a**).

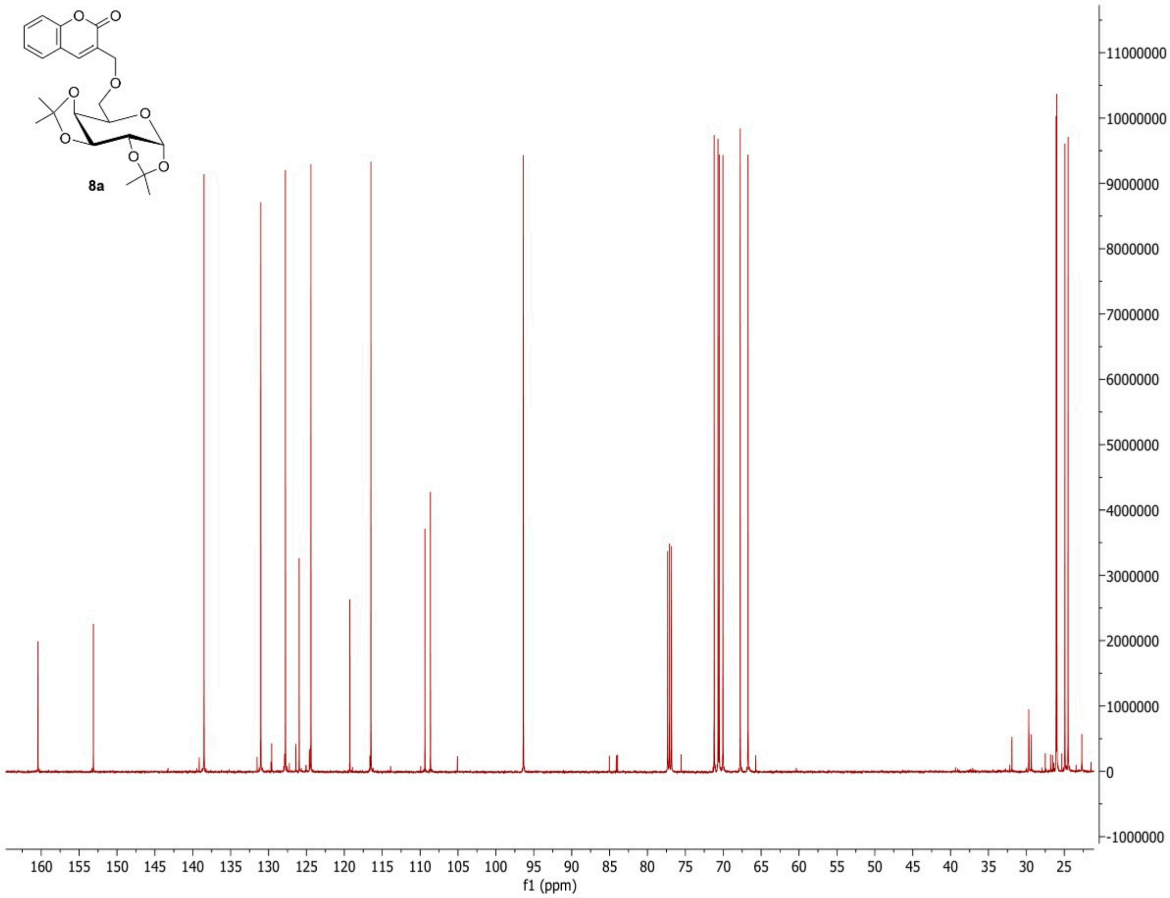

Figure S13. ^{13}C NMR spectra of 6-*O*-(2*H*-chromene-2-one-3-yl-methyl)-1,2:3,4-di-*O*-isopropylidene-α-D-galactopyranose (**8a**).

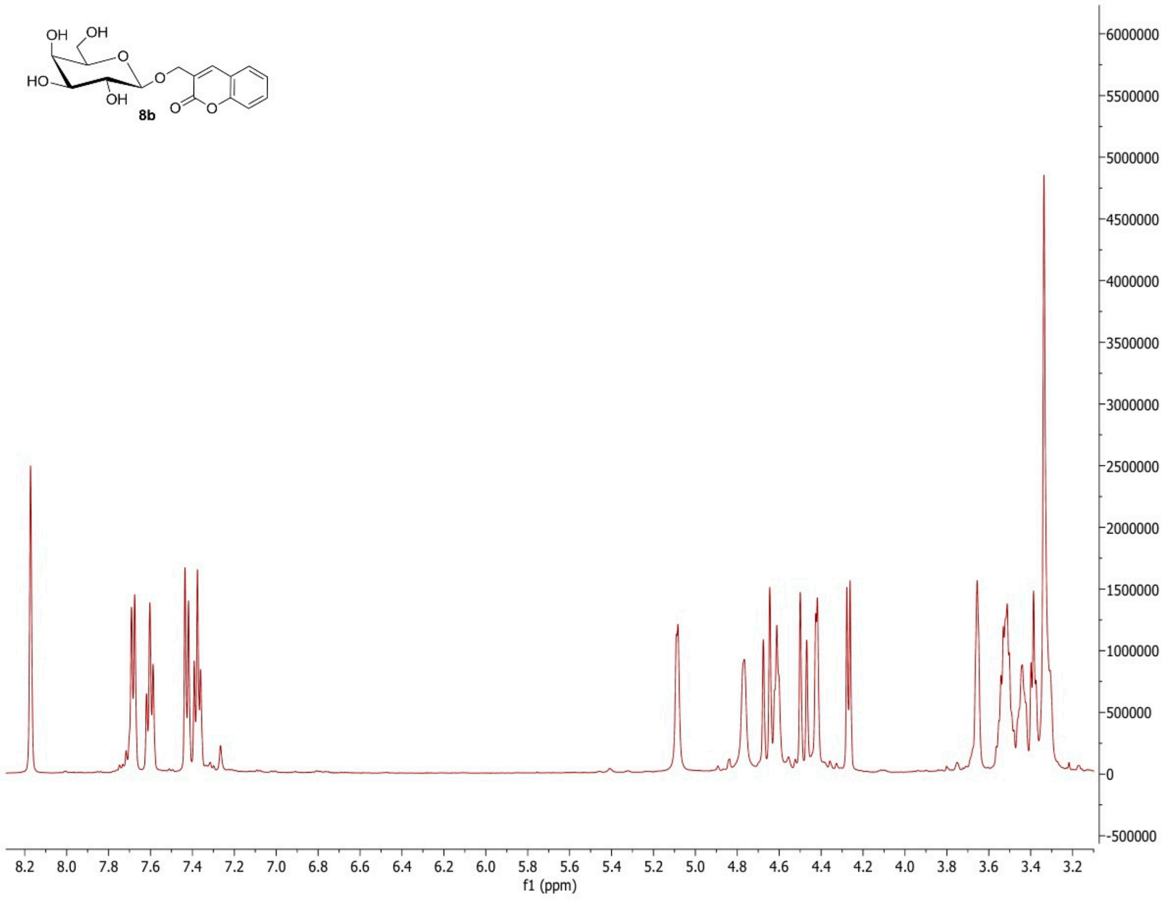

Figure S14. ^{1}H NMR spectra of 1-*O*-[2*H*-chromene-2-one-3-yl-methyl]-β-D-galactopyranoside (**8b**).

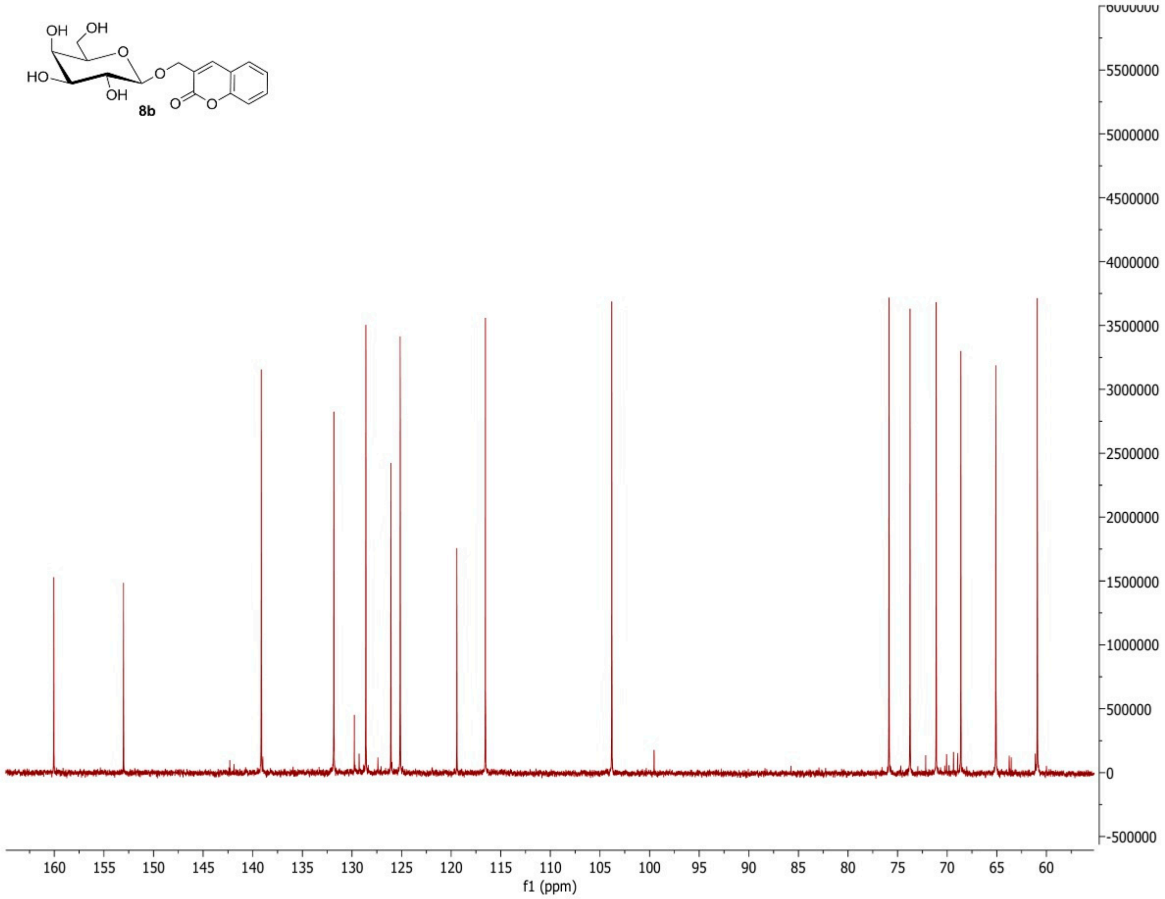

Figure S15. ^{13}C NMR spectra of 1-*O*-[2*H*-chromene-2-one-3-yl-methyl]-β-D-galactopyranoside (**8b**).

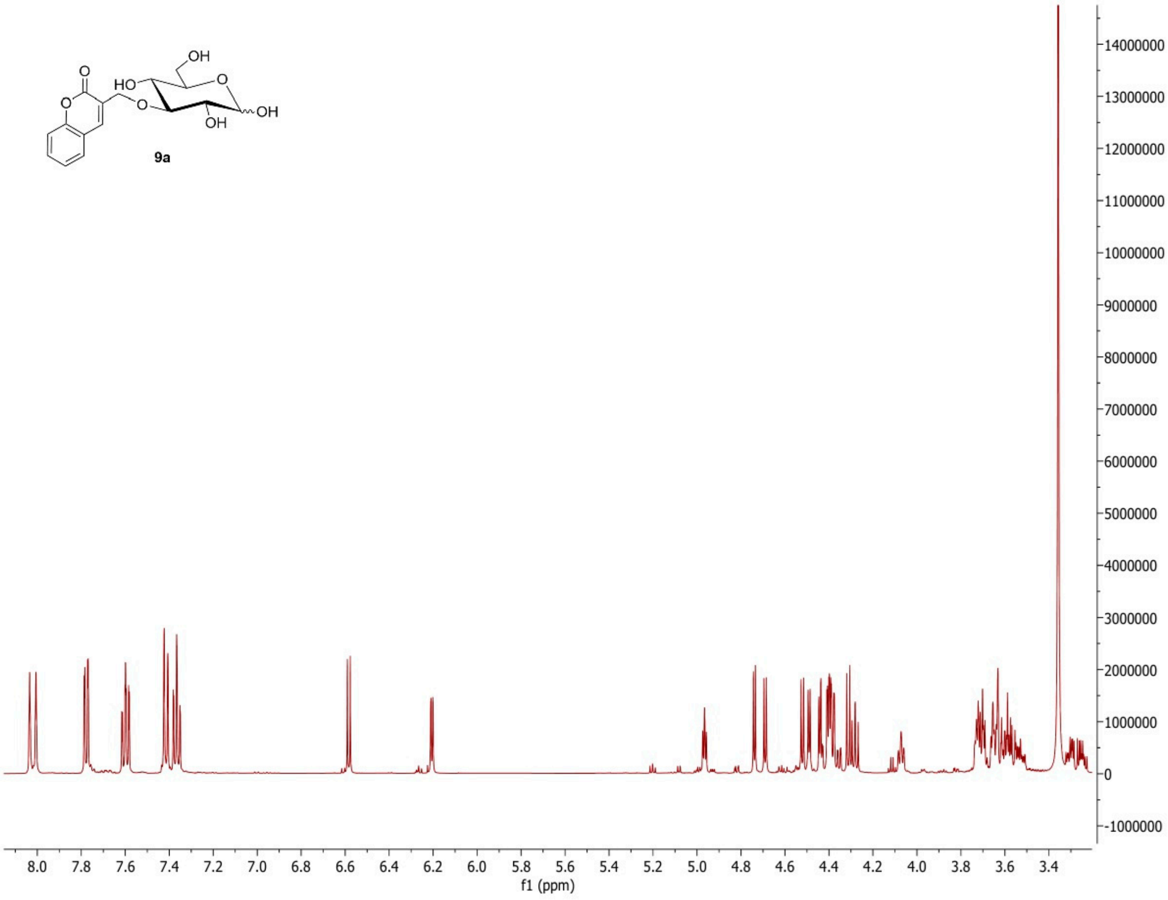

Figure S16. ^{1}H NMR spectra of 3-*O*-(2*H*-chromene-2-one-3-yl-methyl)-D-glucopyranose (**9a**).

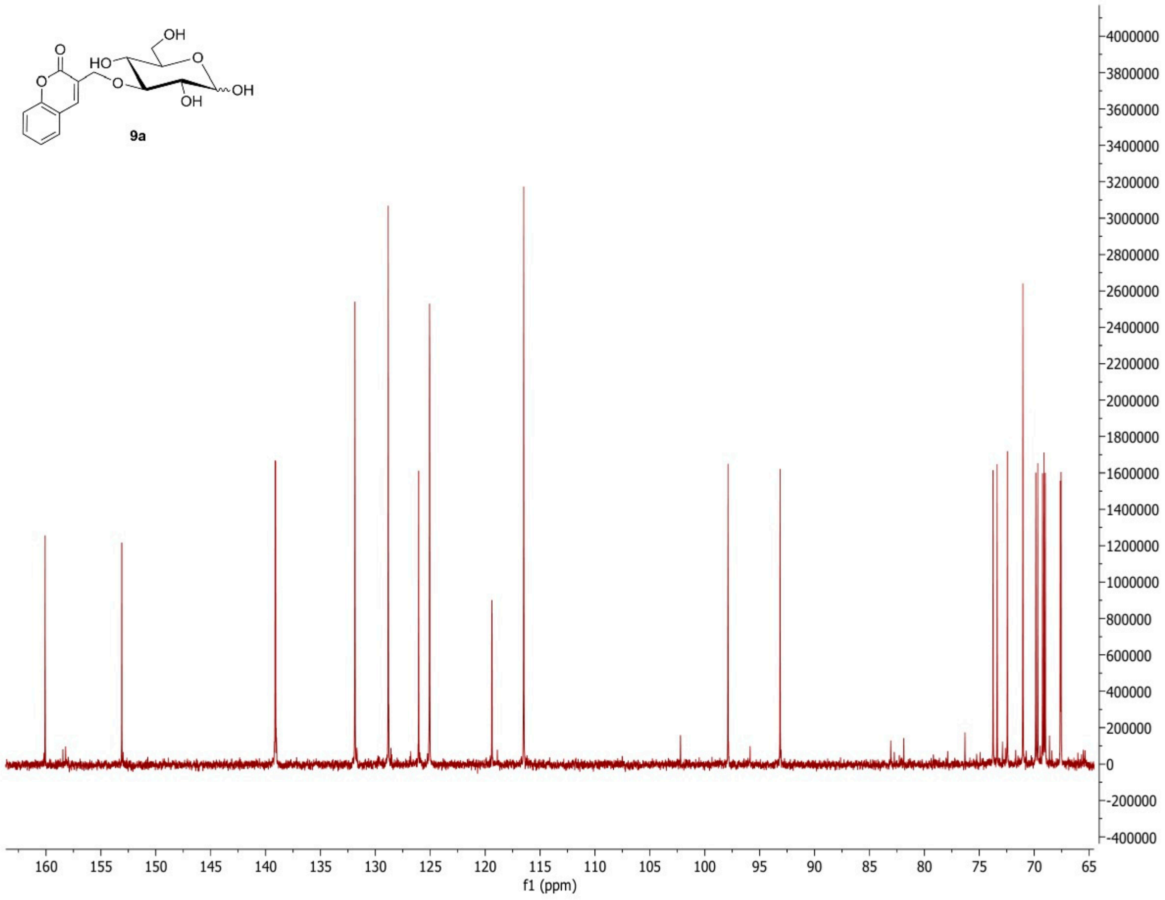

Figure S17. ^{13}C NMR spectra of 3-*O*-(2*H*-chromene-2-one-3-yl-methyl)-D-glucopyranose (**9a**).

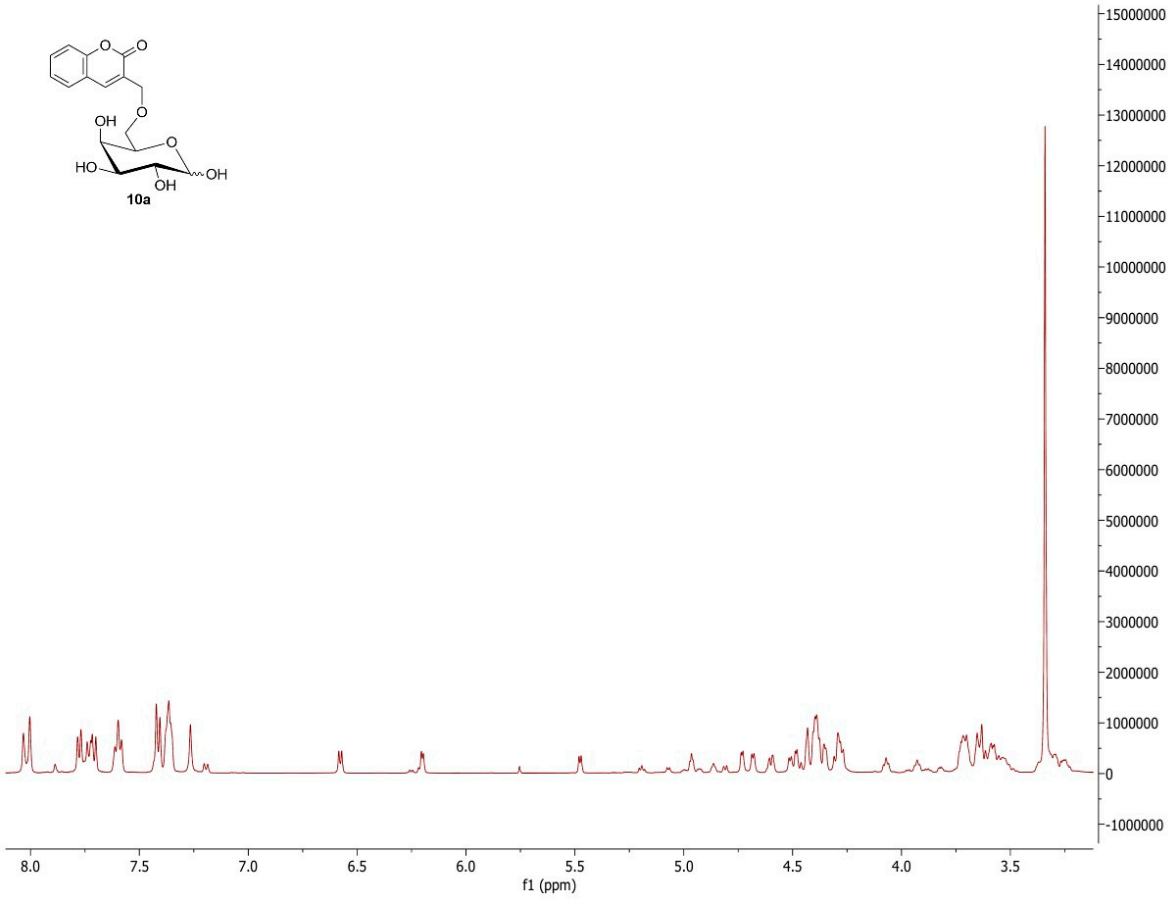

Figure S18. ^1H NMR spectra of 6-*O*-(2*H*-chromene-2-one-3-yl-methyl)-D-galactopyranose (**10a**).

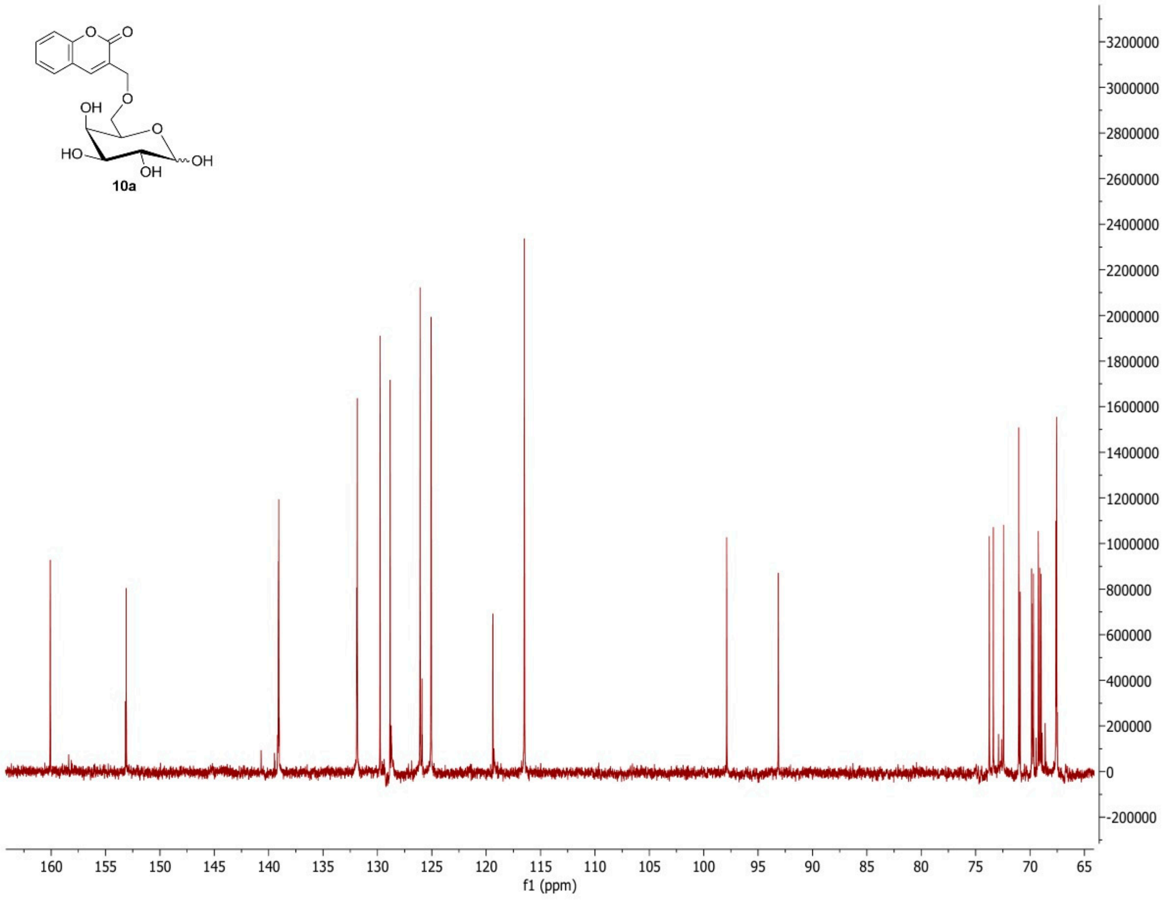

Figure S19. ^{13}C NMR spectra of 6-*O*-(2*H*-chromene-2-one-3-yl-methyl)-D-galactopyranose (**10a**).

Residue – Interaction	%
THR200-VdWContact	98.1
HIS201-VdWContact	94.1
HIS201-HBAcceptor	85.4
THR200-HBAcceptor	85.1
HIS95-VdWContact	81.2
HIS65-VdWContact	81.2
HIS201-Hydrophobic	80.0
LEU199-VdWContact	77.5
HIS68-Hydrophobic	75.9
HIS95-Hydrophobic	69.7
PRO202-VdWContact	60.0
HIS65-HBAcceptor	48.1
GLN93-VdWContact	40.0
VAL63-VdWContact	39.2
SER66-VdWContact	35.8
PRO203-VdWContact	34.9
HIS68-VdWContact	32.4
GLN93-Hydrophobic	25.9
HIS97-VdWContact	21.7
PHE92-Hydrophobic	19.2
TRP6-VdWContact	18.0
PHE92-VdWContact	17.3
LEU199-Hydrophobic	14.2
LEU132-VdWContact	9.2
TRP210-VdWContact	7.6
ALA136-VdWContact	5.9
HIS65-HBDonor	5.4

Table S1a. Protein–ligand interaction fingerprints for the *h*CA I–α-**10a** complex. Lists the residues exhibiting the highest interaction occupancy (%) during the molecular dynamics trajectory. Interactions were categorized as van der Waals (vdW) contacts, hydrogen bond acceptors (HBA), and hydrophobic interactions. Only contacts with an occupancy >5% are highlighted. This analysis was done with Prolif.

**Residue – Interaction**	**%**
GLN93-VdWContact	100.0
GLN93-HBAcceptor	99.5
PHE92-Hydrophobic	96.8
HIS95-VdWContact	92.6
PRO202-VdWContact	92.6
PRO202-HBDonor	88.4
HIS201-VdWContact	86.8
TRP6-VdWContact	85.8
HIS68-VdWContact	78.4
HIS65-VdWContact	74.5
TRP6-HBAcceptor	71.9
HIS65-HBAcceptor	66.8
HIS68-HBAcceptor	63.5
PRO203-VdWContact	51.4
LEU199-VdWContact	50.3
PHE92-VdWContact	49.9
TYR205-Hydrophobic	41.5
LEU132-Hydrophobic	28.5
ALA136-VdWContact	21.4
LEU142-VdWContact	20.4
ALA122-VdWContact	19.4
VAL63-VdWContact	19.1
LEU132-VdWContact	7.2
TYR205-VdWContact	5.7

Table S1b. Protein–ligand interaction fingerprints for the *h*CA I–β-**10a** complex. Lists the residues exhibiting the highest interaction occupancy (%) during the molecular dynamics trajectory. Interactions were categorized as van der Waals (vdW) contacts, hydrogen bond acceptors (HBA), and hydrophobic interactions. Only contacts with an occupancy >5% are highlighted. This analysis was done with Prolif.

Residue – Interaction	%
HIS64-VdWContact	99.8
THR199-VdWContact	99.2
PHE130-Hydrophobic	98.3
GLN92-Hydrophobic	98.0
THR199-HBAcceptor	97.0
HIS64-HBAcceptor	96.3
ASN62-VdWContact	87.1
GLN92-VdWContact	87.0
GLU69-VdWContact	84.3
GLU69-HBDonor	83.9
GLN92-HBAcceptor	79.9
HIS94-VdWContact	74.7
ASN62-HBAcceptor	64.2
PHE130-VdWContact	46.0
ASN67-VdWContact	45.3
VAL121-Hydrophobic	41.6
LEU60-VdWContact	28.9
VAL121-VdWContact	27.3
LEU197-VdWContact	19.2
TRP5-VdWContact	9.0
ILE91-Hydrophobic	5.5

Table S2a. Protein–ligand interaction fingerprints for the *h*CA II–α-**10a** complex. Lists the residues exhibiting the highest interaction occupancy (%) during the molecular dynamics trajectory. Interactions were categorized as van der Waals (vdW) contacts, hydrogen bond acceptors (HBA), and hydrophobic interactions. Only contacts with an occupancy >5% are highlighted. This analysis was done with Prolif.

Residue – Interaction	%
GLN92-VdWContact	98.3
THR199-VdWContact	95.6
THR199-HBAcceptor	95.3
THR198-VdWContact	94.9
LEU197-Hydrophobic	94.4
GLN92-HBAcceptor	93.9
ASN62-VdWContact	89.5
LEU197-VdWContact	87.8
ASN67-VdWContact	82.9
PHE130-Hydrophobic	81.2
THR199-HBDonor	80.6
THR198-HBAcceptor	78.0
ASN67-HBDonor	65.4
ASN62-HBAcceptor	57.4
PRO200-VdWContact	55.0
HIS94-VdWContact	49.2
PRO201-VdWContact	20.6
PHE130-VdWContact	16.9
PRO200-HBDonor	9.8

Table S2b. Protein–ligand interaction fingerprints for the *h*CA II–β-**10a** complex. Lists the residues exhibiting the highest interaction occupancy (%) during the molecular dynamics trajectory. Interactions were categorized as van der Waals (vdW) contacts, hydrogen bond acceptors (HBA), and hydrophobic interactions. Only contacts with an occupancy >5% are highlighted. This analysis was done with Prolif.

Residue – Interaction	%
THR333-VdWContact	100.0
THR333-HBAcceptor	100.0
LEU331-Hydrophobic	98.3
HIS226-VdWContact	91.0
LEU331-VdWContact	84.5
ASN198-VdWContact	83.2
HIS200-VdWContact	75.1
THR332-VdWContact	67.3
PRO334-VdWContact	66.5
ASN198-HBAcceptor	55.5
PRO334-HBDonor	44.9
TRP141-VdWContact	41.9
SER201-VdWContact	36.1
HIS226-HBDonor	35.1
GLN224-VdWContact	34.4
THR332-HBAcceptor	33.1
THR333-HBDonor	29.5
HIS228-VdWContact	21.9
PRO335-VdWContact	19.3
GLN203-VdWContact	18.1
GLN203-HBDonor	16.1
GLN224-HBAcceptor	15.3
VAL253-VdWContact	11.6
HIS200-HBDonor	9.7
TYR143-VdWContact	7.9

Table S3a. Protein–ligand interaction fingerprints for the *h*CA IX–α-**10a** complex. Lists the residues exhibiting the highest interaction occupancy (%) during the molecular dynamics trajectory. Interactions were categorized as van der Waals (vdW) contacts, hydrogen bond acceptors (HBA), and hydrophobic interactions. Only contacts with an occupancy >5% are highlighted. This analysis was done with Prolif.

Residue – Interaction	%
THR333-VdWContact	100.0
THR333-HBAcceptor	100.0
GLN224-VdWContact	99.6
GLN224-HBAcceptor	97.9
ASN198-VdWContact	97.0
LEU331-Hydrophobic	95.8
ASN198-HBAcceptor	93.7
HIS226-VdWContact	86.3
THR332-VdWContact	81.5
LEU331-VdWContact	79.6
TYR143-VdWContact	60.4
GLN203-VdWContact	60.2
HIS228-VdWContact	55.8
TYR143-HBDonor	54.1
GLN203-HBDonor	51.8
SER201-VdWContact	49.7
THR332-HBAcceptor	46.5
HIS200-VdWContact	43.2
THR333-HBDonor	17.9
SER201-HBDonor	17.7
SER201-HBAcceptor	16.6
VAL253-VdWContact	12.0
VAL262-VdWContact	11.0
GLN203-HBAcceptor	5.9

Table S3b. Protein–ligand interaction fingerprints for the *h*CA IX–β-**10a** complex. Lists the residues exhibiting the highest interaction occupancy (%) during the molecular dynamics trajectory. Interactions were categorized as van der Waals (vdW) contacts, hydrogen bond acceptors (HBA), and hydrophobic interactions. Only contacts with an occupancy >5% are highlighted. This analysis was done with Prolif.

**Residue – Interaction**	**%**
THR227-VdWContact	100.0
THR227-HBAcceptor	99.3
GLN117-VdWContact	98.9
LEU225-Hydrophobic	95.3
GLN117-HBAcceptor	95.1
THR226-VdWContact	90.2
LEU225-VdWContact	88.1
HIS119-VdWContact	78.1
THR226-HBAcceptor	77.0
THR227-HBDonor	75.8
ASN92-VdWContact	47.7
HIS94-VdWContact	36.7
SER95-VdWContact	34.9
LYS97-VdWContact	31.1
ASN92-HBAcceptor	27.3
SER161-VdWContact	14.3
TRP32-VdWContact	12.3
SER158-VdWContact	8.9
ALA157-VdWContact	7.8
ASN92-HBDonor	7.6
LYS97-HBAcceptor	5.6
GLN117-HBDonor	5.6

Table S4a. Protein–ligand interaction fingerprints for the *h*CA XII–α-**10a** complex. Lists the residues exhibiting the highest interaction occupancy (%) during the molecular dynamics trajectory. Interactions were categorized as van der Waals (vdW) contacts, hydrogen bond acceptors (HBA), and hydrophobic interactions. Only contacts with an occupancy >5% are highlighted. This analysis was done with Prolif.

Residue – Interaction	%
THR227-HBAcceptor	100.0
THR227-VdWContact	100.0
GLN117-VdWContact	99.5
LEU225-Hydrophobic	98.5
TYR34-VdWContact	95.7
GLN117-HBAcceptor	93.3
LEU225-VdWContact	90.4
TYR34-HBAcceptor	84.2
HIS119-VdWContact	83.3
HIS121-VdWContact	66.4
HIS94-VdWContact	65.6
THR226-VdWContact	64.7
SER95-VdWContact	51.3
HIS94-HBDonor	46.6
THR227-HBDonor	35.3
ASN92-VdWContact	28.7
THR226-HBAcceptor	21.0
SER158-VdWContact	17.4
ALA157-VdWContact	16.4
ASN92-HBAcceptor	12.9
ASN92-HBDonor	11.2
TYR34-HBDonor	8.8
LEU167-VdWContact	7.7
TRP32-VdWContact	5.9
SER161-VdWContact	5.7

Table S4b. Protein–ligand interaction fingerprints for the *h*CA XII–β-**10a** complex. Lists the residues exhibiting the highest interaction occupancy (%) during the molecular dynamics trajectory. Interactions were categorized as van der Waals (vdW) contacts, hydrogen bond acceptors (HBA), and hydrophobic interactions. Only contacts with an occupancy >5% are highlighted. This analysis was done with Prolif.
